# Informal carers’ experience of assistive technology use in dementia care at home: a systematic review

**DOI:** 10.1186/s12877-019-1169-0

**Published:** 2019-06-14

**Authors:** Vimal Sriram, Crispin Jenkinson, Michele Peters

**Affiliations:** Health Services Research Unit, Nuffield Department of Population Health, University of Oxford, Richard Doll Building, Old Road Campus, Oxford, OX3 7LF UK

**Keywords:** Dementia, Assistive technology, Carers, Well-being, Systematic review, Quality of life

## Abstract

**Background:**

Dementia is a health and care priority globally. Caring for persons with dementia is a challenge and can lead to negative psychological, physiological and financial consequences for informal carers. Advances in technology have the potential to assist persons with dementia and their carers, through assistive technology devices such as electronic medication dispensers, robotic devices trackers and motion detectors. However, little is known about carers’ experience and the impact of these technologies on them. This review aims to investigate the outcomes and experience of carers of persons with dementia, who live at home and use assistive technology.

**Methods:**

A systematic search in seven databases and manual searches were carried out using pre-defined inclusion and exclusion criteria to identify studies on carers of persons with dementia involving the use of assistive technology. The search identified 56 publications with quantitative, qualitative and mixed-method designs.

**Results:**

The studies reported positive and negative findings and focused on a wide variety of assistive technology devices. There were large differences in the uses of assistive technology, outcome measures used and the quality of studies. Knowledge and acceptance, competence to use and ethical issues when using assistive technology were themes that emerged from the studies. Carers generally appreciated using assistive technology and their experience of use varied.

**Conclusions:**

The intention of this systematic review is to list and classify the various types of assistive technology used by carers of persons with dementia and explores the positive and negative aspects, knowledge, acceptance and ethical issues in the use of assistive technology by carers of persons with dementia. We recommend the use of a standard and person-centred system of classifying and naming assistive technology devices and systems and for future research efforts in assistive technology to incorporate a family/carer centred model.

**Systematic review registration:**

PROSPERO - CRD42017082268.

**Electronic supplementary material:**

The online version of this article (10.1186/s12877-019-1169-0) contains supplementary material, which is available to authorized users.

## Background

Dementia is a complex acquired brain condition characterised by a decline from a previous level of cognitive functioning with impairment in cognitive domains [1]. Worldwide there are an estimated 50 million people who have dementia and nearly 10 million new cases every year [2]. Informal carers (families, friends and neighbours) provide the majority of care for persons with dementia [3]. Dementia can be overwhelming for carers of persons with dementia and can cause stress from physical, emotional and economic pressures [4–6]. Stretched health and care resources necessitate alternative and innovative ways to providing care for persons living with dementia [7]. Assistive technology (AT) has been suggested as a means to support someone who has dementia and their carers to stay independent and remain in the community [8–11]. AT can be defined as: “any item, piece of equipment, product or system that is used to increase, maintain or improve the functional capabilities and independence of people with cognitive, physical or communication difficulties” [12]. The use of AT by persons living with dementia may by extension also benefit the carer, as it could offer the potential to increase the support to carers and alleviate some of the burden of caregiving [13–16]. AT may assist carers to address the increased level of responsibility whilst caring for a person with dementia [17, 18]. Additionally, carers of someone who has dementia are in the unique position of using their pre-conceived ideas regarding AT to suggest and decide on the access to and use of AT [19], yet very little is known about carers’ experiences of AT use.

## Why it is important to do this review

Currently, AT and Artificial Intelligence driven healthcare solutions are being viewed as a panacea for reducing carer burden [20, 21] and multiple studies are investigating how AT can support people with dementia [22–24]. Carers could be using the AT together with the person with dementia (such as safety alarms) and/or carers could be looking after someone who has dementia, who uses AT independently of the carer. Yet, little is known about the experiences of carers using AT and what impact AT has on carer health and wellbeing [25]. This review, aims to fill the gap in literature that so far has predominantly looked at AT from the perspective of people living with dementia and its use within institutional settings [7, 26, 27] as well as identifying carer wellbeing when using AT. This information could benefit carers and persons with dementia considering AT solutions for use at home, help healthcare professionals who prescribe and set up AT solutions, as well as developers/inventors of AT.

## Review aim and questions

This review aims to:

1. Identify the types and uses of AT in dementia;
2. Describe the effectiveness of AT for outcomes (including burden, well-being and quality of life) of carers of people with dementia living at home;
3. Describe carers’ experiences of AT use in dementia;
4. Determine the aspects of AT that are valued and work well for carers by integrating (2) and (3) as above.

## Methods

The review protocol was registered with the international prospective register of systematic reviews PROSPERO (CRD42017082268). The Preferred Reporting Items for Systematic Reviews and Meta-Analyses (PRISMA) checklist is included as Additional file 4.

### Types of studies

Quantitative, qualitative and mixed method study designs were included. Letters to the editor, abstract and conference proceedings, book reviews, study protocols and theses/dissertations were excluded. We did not include other reviews but checked references within identified existing reviews on dementia, informal carers and AT to ensure that all relevant studies had been located. Due to funding constraints, only studies in English language or those translated to English language were included.

#### Evaluation of effectiveness

We included all randomised and controlled trials that compared AT for carers of someone who has dementia to those not provided with the AT, and who received usual care. We also included observational and cohort studies.

#### Evaluation of experience

We included studies that used qualitative methods of data collection and analysis, either as a stand-alone qualitative study or as part of a mixed-method study.

### Types of participants

Studies that included carers who provide unpaid care for a person living with dementia at home were included. Providing care is defined for the purposes of this study as ‘supporting a person with dementia physically, emotionally, financially or socially’ and care could be provided by a relative, a friend or a neighbour. There were no restrictions regarding gender, living arrangements or ethnic background. Studies reporting on carers who provide support to a person living with dementia receiving care in hospital and/or long-term institutions and carers younger than 18 years and formal/paid carers were excluded.

### Types of assistive technology

For this review, studies that evaluate AT use in dementia involving carers were included. AT was defined as ‘any advanced electronic equipment, which can be used to enhance support and care, act as a prompt for intervention by carers, monitor welfare and assist in communication and leisure activities for a person with dementia’. This AT can be standalone (e.g. Tablet computers) or be part of an integrated system (e.g. GPS and sensor trackers) and can be stationary or mobile. As the focus of most research studies invariably is on the person living with dementia, any study that reported on effects or experiences of AT use on carers were included. Studies that reported only on AT use for people with dementia without including carers were excluded, as were studies that focus only on electronic therapeutic interventions that are not AT (e.g. computer-based education or support for carers).

### Types of study outcome measures

The search was not limited to specific types of outcome measures and included carer self-reported outcome measures of burden; quality of life; and well-being; and self-reported or researcher observed experiences of usefulness; benefits and disadvantages of AT and impact on carer /person living with dementia relationship.

### Search strategy

The search strategy was developed in collaboration with a Bodleian medical library librarian at the University of Oxford.

Searches were carried out on:

#### Databases

Including MEDLINE (Ovid) from 1946 to June 2018; EMBASE from 1974 to June 2018; PsycINFO from 1806 to June 2018; AMED 1985 to June 2018; CINAHL from 1981 to June 2018; Database of Abstracts of Reviews of Effects (DARE), OT seeker and The Cochrane Library of Systematic Reviews. The search included studies within ALOIS (from inception to June 2018).

#### Unpublished literature

The International Standard Randomised Controlled Trials Number (ISRCTN) registry [28] and the National Institutes of Health Clinical Trials Database [29] were searched for information on unpublished ongoing trials. Searches within these databases were used to identify additional studies and authors to contact for full text reports.

#### Manual searches

We also conducted manual searches of reference lists to identify relevant research studies.

Details of the full search, with search strategies and the number of records identified in each database are included in Additional file 1.

### Screening

Electronic search results were downloaded into Covidence software [30] (an online digital platform that streamlines the production of systematic reviews and allows screening and data extraction between collaborating reviewers) as *.ris* files. Duplicates were removed using the software. Authors VS and MP independently screened all titles and abstracts for eligibility against the inclusion/exclusion criteria. For studies that had insufficient information from the title and abstract, full text articles were retrieved to determine inclusion. Studies marked for possible inclusion underwent a full-text review. At full-text review, when both VS and MP agreed that a study did not meet the full eligibility criteria, the study was excluded. CJ was consulted when VS and MP did not agree on a study. Discrepancies were resolved by mutual discussion.

### Data extraction

A bespoke data extraction form (Additional file 2) developed by all the authors was used and initially piloted on a sample of studies to refine the form. Data from the studies were logged using Microsoft Excel 2016. There were no deviations from the published protocol.

#### Effectiveness

Data extraction items from quantitative studies were based on the recommended items from the Cochrane handbook for systematic reviews of interventions [31]. Information on citation including authors, date of publication, study design, duration, number of participants, participant gender, age, ethnicity, country where the study took place, relationship status to the person living with dementia, types and use of the AT, outcome measures used, time points of data collection, missing participants and key conclusions from the study authors were extracted.

#### Experience

In addition to collecting information from qualitative studies on citation, author details, study design, duration, and participant information, country and time points when information was collected, VS extracted data based on study authors’ commentaries and conclusions [32, 33]. MP and CJ checked extracted data for accuracy and completeness. Disagreements and clarifications were resolved by discussion among the authors.

## Results

**Fig. 1:**
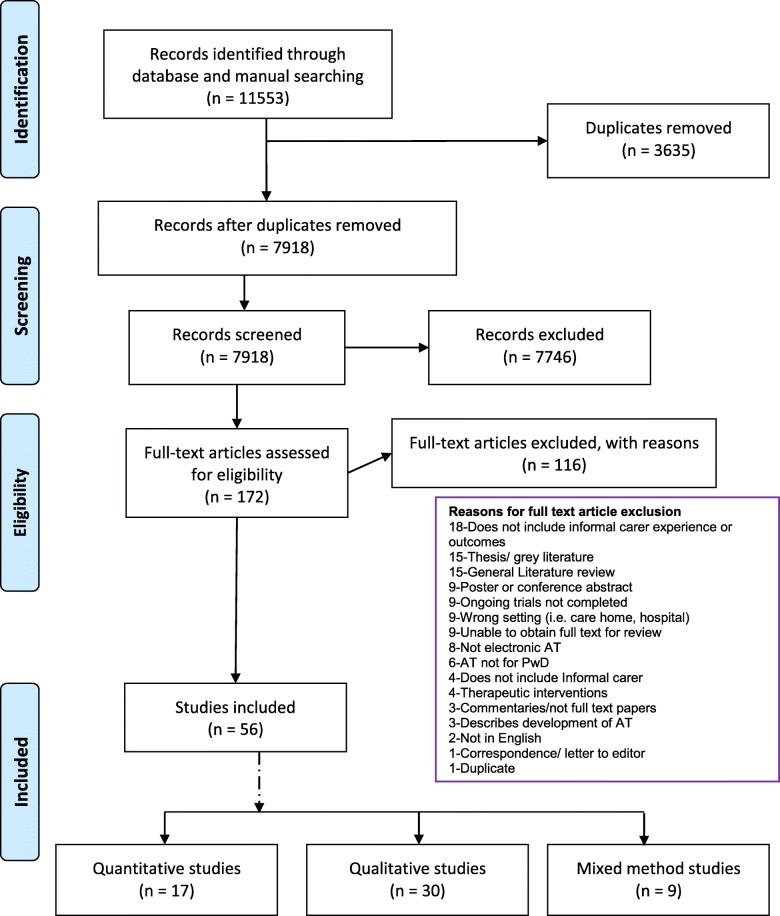
PRISMA flow diagram of study selection

The first search was carried out in December 2017 and repeated in June 2018. A check for duplicate records was carried out electronically. To confirm results gained from Covidence [30] an additional screening using reference management software Mendeley [34] was undertaken. From the 11,553 records retrieved from database search 3635 were removed as duplicates. The titles and abstracts of the 7918 retrieved records were independently screened by VS and MP. A total of 7746 records were excluded (including further duplicate records) and full-text articles for the remaining 172 records were independently assessed for inclusion based on full texts by VS and MP. Fifty-six papers met the inclusion criteria and were included in this review for data extraction. Reasons for exclusion of the full-text papers were documented and are listed separately (Fig. 1).

### Included studies

Of the 56 included publications, 30 were qualitative, 17 quantitative and 9 mixed-methods reporting on a total of 50 studies from 19 countries. There were 2 Randomised Controlled Trials (RCTs) and 1 Controlled Clinical Trial. The publications were from 2000 to 2018, reporting on findings from 2016 carers (660 men and 1165 women, where gender was reported) and 84 types of AT. Carers’ age ranged from 19 to 91 years, with 13 publications not reporting an age range for participants. Several methods were used for data collection including interviews (32), surveys (14), observations (8), focus groups (7), questionnaires (6), diary/log entries (4) and video recording and email and blog reviews (1 each), with 19 studies using more than one method for data collection. Seven studies [35–42] reported on ethnic backgrounds of participants which were predominantly from white backgrounds alongside Hispanic, African American, Asian and ‘other’ backgrounds. Most studies reported the severity and type of dementia, without specifying a classification system; for ease of use, we have followed the 3-stage classification system of clinical dementia rating assessment [43]. Where reported in the studies, most studies involved people with dementia who had mild or moderate dementia. The carer relationship to the person with dementia ranged from spouses, siblings, children, daughter/son-in-law, nieces and nephews, grandchildren, neighbours and friends. Some studies included paid carers and participants who lived in long-term care facilities alongside carers of someone who has dementia living at home. The results described in this review relate only to family carers and persons with dementia who lived at home from those studies.

### Quality of included studies: appraisal of included studies

As this review involved quantitative, qualitative and mixed-method studies, the Mixed Methods Appraisal Tool [18, 44, 45] for assessing quality of included studies was used. MMAT scores are provided (Additional file 3) for the included publications. The score is a subjective appraisal of a study’s methodological quality. For qualitative and quantitative studies, the percentage of criteria met is stated. For mixed-methods studies, the overall score cannot exceed the lowest score of a component, so if one part received 100% but the other 50%, the overall score would be 50%. This means it would be possible for a study to have a strong quantitative section and a weaker qualitative section, or vice-versa, but the overall score would be low, suggesting the study might be less valuable [18]. The included studies were critically evaluated by VS and discussed with MP and CJ with discrepancies resolved through discussion. A majority of the included publications scored over 50% on the MMAT score with six of the qualitative studies [46–51] scoring highly for due consideration of results in context and for researchers’ own influence on data collection and interpretation of results. The RCTs [35, 36] and the controlled clinical trial [37] scored poorly on allocation concealment and blinding and the RCT pilot trial [36] also had a high attrition rate. While not ignoring the strengths and weaknesses of the studies, we have included all studies, to answer the questions for this review and add to the richness of our findings. Results are presented in line with the questions that this systematic review set out to explore. Characteristics of the included papers are presented in Table 1 and additional details are available in Additional file 3.

**Table 1:** Characteristics of included studies

Qualitative studies								
No	Authors Date	Country	Participants	Age range	Study Design	Theoretical Framework	Assistive Technology	Data collected from
1	Altus DE et al. [52] 2000	USA	2- Spouses (2 men)	Not reported	Interviews	Case study	GPS tracker/Mobile locator	Participant reflections and diary of incidents
2	Cahill S [46] 2007	Ireland	20 – Spouses + Children + Sibling + Daughter-in-law + Friend (5 men; 15 women)	34–79	Semi-structured interviews	Thematic Analysis	Automatic night and day calendar; lost item locator; automatic night lamp; gas cooker device; picture button telephone;	Participant reflections
3	Starkhammar S et all [53] 2008	Sweden	14 Spouses + Daughters (5 men; 9 women)	Not reported	Interviews and Observations	Grounded Theory	Stove timer	Participant reflections
4	Faucounau V et al. [54] 2009	France	1 – Spouse (1 woman)	68	Interviews	Single dyad case study	GPS tracker	Participant reflections
5	Spring HJ [40] 2009	USA	14 – Spouses + Daughters + Grand daughter (1 men; 13 women)	38–86	Semi-structured interviews	Full conceptual description- grounded theory	In-home night time monitoring system	Participant reflections
6	Landau R et al. [55] 2010	Israel	36 – Spouses + Children (12 men; 24 women)	Not reported	Focus groups	Thematic framework approach	GPS electronic tracking device	Participant reflections on vignettes
7	Powell J et al. [41] 2010	England	34 – Spouses + Children + Grandchildren + Friend (12 men; 22 women)	23–91	Semi-structured interviews	Framework approach	Movement sensors; cameras; automatic water and gas switch off devices; tracking devices	Participant reflections on vignettes
8	White EB et al. [56] 2010	England	10 – Spouses + Son+ Daughter (4 men; 6 women)	44–73	Interviews and survey	Grounded Theory	GPS tracker	Participant reflections and Machin carer strain scale (modified)
9	Rosenberg L et al. [57] 2011	Sweden	4 – Son +Daughters +Neighbour (1male; 3 women)	55–78	Observations and In-Depth interviews	Grounded Theory	Night and Day Calendar, Forget-Me-Not Calendar, Memory Message, MeDose watch	Participant reflections
10	Olsson A et al. [58] 2012	Sweden	14 Spouses (6men; 8 women)	62–89	Interviews	Not reported	Safety alarm, bed alarm, door alarm, passage sensor, electronic tracking device, cooker monitors, talking cazette, picture button telephone, memory aid, special remote control	Participant reflections
11	Rosenberg L et al. [59] 2012	Sweden	16 – Spouses + Son +Daughter+ Neighbour (5 men; 11 women)	45–78	Interviews and Focus groups	Grounded Theory	GPS monitor, electronic pill dispenser	Participant reflections
12	Martin S et al. [60] 2013	Northern Ireland	8 (Gender not reported separately)	Not reported	Semi-structured interviews	Phenomenological approach	NOCTURNAL - Sensors, communication devices, tablet PC	Participant reflections
13	Nijhof N et al. [61] 2013	Netherlands	14 – Spouses + Family + Friends (Gender not reported)	Not reported	Semi-structured interviews	Not reported	ADLife - gateway with alarm button, sensors	Participant reflections
14	Olsson A et al. [62] 2013	Sweden	5 Spouses (2men; 3 women)	62–68	Participant Observation	Ethnographic approach	Passive Positioning Alarm	Participant experience
15	Riikonen M et al. [47] 2013	Finland	25 – Spouses + Daughters + Brothers + Son-in-law + Niece (12 men; 13 women)	Less than 65	Unstructured interviews and participant observations	Thought entity	Cameras, movement detectors, electronic medication reminder, photo memory telephone	Participant reflections
16	Hastall MR et al. [63] 2014	Germany	17 - Spouses + Children (4 men; 13 women)	38–91	Semi-structured interviews	Thematic Analysis	Information retrieval system; Video communication system; sensors	Participant reflections on vignettes
17	Jentoft R et al. [64] 2014	Norway	9 – Son + Spouses +Daughters +Mother (4 men; 5 women)	19–89	In-depth interviews and Observations	Social situated learning	Simple remote control for TV	Participant reflections
18	Meiland F et al. [65] 2014	Netherlands and Germany	13 (Gender not reported separately)	Not reported	Interviews and Focus group	Not reported	COGKNOW day navigator; sensors and sensor network	Participant reflections on development of AT + Vignette
19	Milne H et al. [66] 2014	Scotland	16 - Spouses + Sister + Son + Daughters + Son-in-law + Daughter-in-law (7men; 9 women)	Not reported	Interviews (part of observational mixed methods study)	Thematic analysis with constant comparison	GPS devices - worn as watches, pendants or carried in pockets and bags	Participant reflections
20	Burstein AA et al. [67] 2015	USA	34 - Spouses + Children + Grandchildren + Friend + Neighbour + Sibling + Niece + Daughter-in-law + Cousin (6 men; 28 women)	43–76	In-depth interviews	Not reported	Sensor technology, GPS tracking device. Plus emerging technology - robotic therapy seal, GPS tracking system; social contact system, health monitoring system	Participant reflections on awareness of technology
21	Gibson G et al. [10] 2015	England	26 - Spouses and Daughters (Gender not reported separately)	49–82	Interviews	Thematic analysis, constant comparative method	Community alarms and telecare; GPS location monitoring devices, signage, reminiscence tools, clocks to aid orientation, simplified telephones with pictures, pill dispensers	Participant reflections
22	Arntzen C et al. [48] 2016	Norway	14 - Spouses +Children +Parent Gender not reported)	19–89	In-depth interviews and Observations	Phenomenological conception of ‘lived body’	Sensors, timers, tracking device, cooker alarm, timer on coffee machine, automatic day and night calendar, simple remote control for television, electronic door lock, item locator, simple mobile phone, automatic day and date calendar, speaking arm-wrist watch, message box reading out a message when activated, memory clock, medicine dispenser with alarm	Participant reflections
23	Grigorovich A et al. [68] 2016	Canada	20 – Sons + Son-in-law (20 men)	25–66	Semi-structured interviews	Naturalistic enquiry, qualitative descriptive design	Cameras, baby monitors, skype	Participant reflections
24	Newton L et al. [69] 2016	England	26 (Gender not reported separately)	49–82	Interviews	Not reported	Community alarms and telecare; GPS location monitoring devices, signage, reminiscence tools, clocks to aid orientation, simplified telephones with pictures, pill dispensers	Participant reflections
25	Ekstrom A et al. [70] 2017	Sweden	1 – Spouse (1 male)	Not reported	Video recording and observation	Talk-in interaction	Tablet computer	Length and domains of conversation
26	Evans N et al. [49] 2017	England	6 – Daughters +Spouses +Daughter-in-law (6 women)	48–62	Interviews and Focus groups	Interpretative Phenomenological Analysis	Calendar	Participant reflections
27	Hassan L et al. [71] 2017	England	4 (Gender not reported)	Greater than 65	Focus group	Not reported	Wearable sensors	Participant reflections and discussion on vignettes
28	Holthe T et al. [50] 2017	Norway	13 – Spouses + Daughter + Mother + son (7 men; 6 women)	19–89	Interviews	Epistemology of coping	stove timer, timer to coffee machine, GPS, automatic calendar, simple remote control to TV, Electronic door lock, object locator, simple mobile phone, digital calendar with remote control, talking wrist watch, medicine dispenser with alarm, memory clock, message box connected to coffee machine	Participant reflections
29	Lorenz K et al. [72] 2017	England	7 – Sons + other carers (Gender not reported)	Not reported	Email and blog reviews	Not reported	Baby monitors, talking motion sensors, personalised recorded messages, cameras	Reports on personal evaluation of technology
30	Wang RH et al. [51] 2017	Canada	10 – Spouses + Son + Daughters (4 men; 6 women)	36–78	Semi-structured interviews	Thematic analysis	Assistive robot - Ed - personal computer; microphones, LCD screen, speakers and web cameras	Participant reflections based on observations
Quantitative studies								
No	Authors Date	Country	Participants	Age range	Study Design	Assistive Technology	Measures	Validated measures
31	Gitlin LN et al. [35] 2010	USA	63/73 [Experimental/Control] – Spouse + Others (13 Men; 50 women – Experimental group) Dropouts not reported	53–79	Randomised controlled trial	Medication dispensers, video cameras, motion detectors, lost item finders	1. Use of device (yes/no). 2. Extent to which helpful (1 = not helpful, 2 = somewhat helpful, 3 = very helpful)	No
32	Rowe MA et al. [36] 2009	USA	26/27 [Experimental/control] – Spouses + Daughter + Son + Granddaughter (7/4 men; 19/23 women). Dropouts = 10 in each group	38–86	Pre test-Post test repeated measures Randomised pilot study	Night Monitoring System	1. System reliability. 2. Satisfaction with the NMS. 3. Nighttime injuries. 4. Unattended exits from home. 5. Mechanisms to manage nightime activity. 6. Baseline variables including short version of Zarit Burden Interview; The neuropsychiatric Inventory	1–5 = No. 6. Zarit Burden Interview = Yes; Quebec User Evaluatio of Satisfaction with Assistive Technology Questionnaire = Yes
33	Rowe MA et al. [37] 2010	USA	26/27 [Experiemental/control] – Spouses + Daughters + Sons (6/3 Men; 18/22 Women). Dropouts = 4 in each group	38–86	Pre test-Post test Controlled Clinical Trial	Night Monitoring System	1. Caregiver distress about night time activity 2. Sleep diary 3. actigraphy	1. Generated for this study (10-point Likert-type scale. 2. Subjective 5-point scale 3. Analysis of sleep-wake cycles
34	Olsson A et al. [73] 2015	Sweden	3 – Spouses (3 women)	72–74	Three - Experimental single case studies (A1B1A2B2)	Passive Positioning Alarm	1. Percentage of days with independent outdoor activities. 2. Spouse’s worry concerning PwD’s independent outdoor activities (1–10 not worried at all to extremely worried). 3. General well-being for spouses (0–10 not well-being at all to extremely good). 4. Caregiver burden scale (1 not at all to 4 often).	1–3 = No; 4 = published for stroke patients
35	Pot AM et al. [74] 2012	Netherlands	33- Spouses + Children (2 Men; 26 Women) Incomplete = 5	> 63	Pre test-Post test Trial	GPS tracking device	1. overall global impression of device from 1 to 10. 2. Structured questions for use of the device from totally agree, totally disagreem agree and disagree. 3. Accepatability using Agree, neutral, disagree. 4. Self-perceived pressure from informal care scale	1–3 = No; 4 = published
36	Kinney J M et al. [38] 2004	USA	19 – Spouses + Son + Daughter + Sister + Great Niece. (8 men; 11 women). Incomplete = 6.	36–82	Survey and exit interview	Smart home management system (Xanboo); cameras and sensors, cell phone	1. Family obligation, competence, role captivity, loss of self, overload, expressive support in caregiving. 2. Retrospective time budget. 3. Exit interview questions	No
37	Duff P et al. [75] 2007	Ireland, England, Finland, Lithuania Norway	127 (Gender not reported) Incomplete = 47	Not reported	Before and After Survey	Calendar, Lamp, Gas cooker monitor, locator, picture telephone, medicine reminder	1. Usefulness. 2. Levels of satisfaction. 3. Recommend device to a friend. 4. Willing to pay for device. 5. Carer wellbeing score	1-4 = No. 5 = Yes
38	Rialle V et al. [76] 2008	France	350 – Spouses + Siblings + Relatives + Other (89 men; 181 women) Incomplete = 80	54–98	Cluster sample survey	Smart home technologies - fall sensor; oral call identification; video surveillance; tracking device; robot care; video conferencing	1. Questionnaire in three sections a. general information; b. Current skills and practice and viewpoints on specific technologies; c. Economical effort and support. (not at all, little, moderately, very much). Included ‘mini’ Zarit score	No
39	Landau R et al. [77] 2009	Germany and Israel	69 – Spouses + Sons + Daughters + Other family members (16 men; 53 women)	Not reported	Survey	GPS tracking device	1. Attitude towards use of electronic tracking questionnaire - 31 items on 4-point Likert scale from do not agree at all to very much agree. 2. Who should make decisions regarding use of GPS device - 4-point Likert scale. 3. Six Items adapted from Quebec User Evaluation of Satisfaction with Assistive Technology scale	No
40	Chen Y-C et al. [78] 2012	Taiwan	3 – Spouses (1 man; 2 women)	28–86	Survey	Electronic GPS	1. Lost seeking devices used. 2. Wandering behaviour. 3. Functions in lost seeking devices. 4. Faults in lost seeking devices. 5. Importance of the information 6. importance of purchase consideration.	No
41	Alwin J et al. [79] 2013	Sweden	47 – Spouses + Children (16 men; 31 women)	46–77	Survey	Easy-to-use telephones, door alarms, alarms and emergency transmitters	1. Carers of Older People in Europe (COPE index). 2. Patient perspective on Care and Rehabilitation process instrument (POCR). 3. How do you value the significance of the intervention? (1 = of no significance, 5 = of greatest possible significance)	1 = published. 2 = Published but modified for this study. 3 = No
42	Lim F S et al. [80] 2013	Australia	24 – Family + Friend (3 men; 21 women) Dropouts = 3	34–85	Before, during and after survey	iPad	1. Previous experience of use of technology. 2. PwD using device independently. 3. Carer’s perspectives on usefulness of the device.	No
43	McKenzie B et al. [81] 2013	USA	60 (7 men; 53 women) incomplete = 1	Not reported	Before and after survey	Motion sensor with remote alarm, wireless camera with handheld liquid crystal display night vision, proximity range alarm, wireless home security system, medication organiser, locating technologies, bed occupancy sensor, touchpad key locks, window alerts, water detector alert	1. Safety assessment Scale. 2. Caregiver Vigilance Scale. 3. Peace of Mind scale. 4. Sleep Disorders inventory	Yes
44	Schulz R et al. [39] 2016	USA	512 – Spouses + Sons +Daughters + Other family members + Friends (251 men; 261 women) Incomplete = 75	18–64	Survey	Emergency response system; sensors;	1. General technology attitudes −10-point scale.	No
45	Kamimura T [82] 2017	Japan	5 – Spouse + Daughter + Daughter-in-law (Gender not reported)	52–65	Survey	Automatic Medication Dispenser	1. Carer Burden (1 = no burden; 2 = little burden; 3 = mild burden; 4 = moderate to severe burden).	No
46	Korchut A et al. [83] 2017	Poland Spain	81- (26 men; 54 women) incomplete = 1	Not reported	Survey	Robotic assistants	1. Social acceptance. 2. human-robot interaction	No
47	Megges H et al. [84] 2017	Germany	18 – Spouses + daughters (10 men; 8 women) Dropout = 1	41–78	Before and after survey	Mobile locating system with GPS	1. Zarit burden interview. 2. General self-efficacy scale. 3. User diary. 4. How satisfied they were with the prototype (1 = not at all satisfied to 4 = very satisfied)	1,2 = Yes. 3,4 = No
Mixed Methods Studies								
No	Authors Date	Country	Participants	Age range	Study Design	Assistive Technology	Measures	Validated measures
48	Topo P et al. [85] 2007	Lithuania Norway Ireland United Kingdom Finland	50 - Spouses + Children + Grandchildren + Friends. (4 men; 21 women). Incomplete = 25	29–84	1. Burden of care questionnaire 2. Interviews	Night and Day Calendar	1. The need for care and treatment. 2. Use of services and quality of life. 3. Greene’s Relative Stress Scale	1,2 = No. 3 = Published
49	Meiland F et al. [86] 2012	Netherlands Ireland Sweden	41 – Spouses + Children (25 men; 16 women)	23–79	Pre test-Post test Questionnaires 2. Semi-structured interviews. 3. Diary. 4. Observations	COGKNOW Day Navigator (CDN) - touchscreen, mobile device, home based sensors, actuators	1. Short Sense of Competence Questionnaire. 2. One question regarding overall judgement on QoL of the carer	1 = Yes. 2 = No.
50	Nijhof N et al. [87] 2013	Netherlands	16 – Spouses + Sons + Daughters (6 men, 10 women)	35–79	1. Log file of system use. 2. Interviews	PAL4 BV - Agenda for the day, diary, two-way video contact, memory games to play, Music and movies, information on dementia and the village they live in	1. Log files - number of clicks and buttons pressed on the touch screen. 2. interviews on uptake and impact with carers	No
51	Mehrabian S et al. [88] 2014	France	30 – Spouses + Children (10 men; 20 women)	54–74	1. Survey 2. Semi-structured interviews.	Telecare system - sensors, videoconferencing, detecting emergencies, cognitive stimulation exercises. Medicines and task reminders	1. Questionnaire on use and usefulness. 2. Interview questions	No
52	Lewis V et al. [89] 2015	Australia	59 – Spouses + Others (16 men; 35 women). Incomplete = 8	30–70	1. Pre test-Post test self-report questionnaire. 2. Diary. 3. Semi-structured phone interviews.	MP3 player	1. Kessler-10 measure of psychological distress −1 = none of the time to 5 = all of the time. 2. General health question-one item. 3. Life satisfaction questions - 1 = very dissatisfied to 11 = very satisfied. 4. Family caregiver self-efficacy scale - 1 = not at all certain to 10 = very certain. 5. Caregiving and stress measure. 6. Self-care and healthy behaviours.	Yes
53	Hattink B J et al. [90] 2016	Germany Netherlands Belgium	17/15 [Experiemental/Control] – Spouses + Children + Other (7/6 men; 10/8 women) Dropouts = 9/9	29–85	1. Pre test-Post test control group design with matched groups (Netherlands, Belgium). Randomised Controlled Trial (Germany). 2. Focus Groups	Integrated Rosetta system: Elderly Day Navigator + The early Detection System + Unattended autonomous surveillance system	1. Usefulness and user-friendliness. 2. Short sense of competence questionnaire.	1 = No. 2 = yes
54	Navarro R F et al. [91] 2016	Mexico	3 – Spouses + Daughter (3 women)	43–66	1. Questionnaires 2. Diary. 3. interviews	Assisted cognition system - touchscreen reminders for the day, current date and time, tracking	1. Caregiver burden - Zarit Burden Interview. 2. Caregiver self-efficacy - revised Scale for caregiving Self-Efficacy	Yes
55	Liu L et al. [92] 2017	Canada	46 - Spouses + Children + Friend + Daughter-in-law (11 men; 35 women)	41–71	1. Pre test-Post test questionnaires2. Focus groups	GPS	1. Questionnaire - 1 = strongly disagree to 5 = strongly agree. 2. Zarit Burden Scale. 3. Focus groups.	No
56	Tyack C et al. [42] 2017	England	12 (2 men, 10 women)	48–77	1. Quasi-experimental repeated measures. Questionnaire. 2. Semi-structured interviews	Tablet Computer	1. Quality of Life - Alzheimer’s Disease (QoL-AD) scale. 2. Semi-structured interviews	Yes Thematic analysis

### Data synthesis

As the included studies were a mixture of quantitative, qualitative and mixed-methods studies, we completed a narrative synthesis of the evidence [32, 33, 93–95]. The narrative synthesis aims to present a descriptive summary of findings across the included studies and themes relevant to the aims of this review, such a synthesis can produce new insights and understanding from different aspects and provides a more informed view of carer experience with AT. Lins et al. describe that when “descriptive qualitative studies that are characterised by ‘thin descriptions’ are available, an aggregative method is more appropriate; if the identified evidence includes a high proportion of in-depth qualitative studies characterised by ‘thick descriptions’, an interpretative method can be applied” [96]. Since all of the qualitative studies in this systematic review had ‘thin descriptions’ available we used an aggregative method for qualitative synthesis.

We followed the method of Timulak [97] for qualitative data synthesis. We transferred data from the extracts of the included studies into data analysis software NVivo Version 12 [98]. The first step of the analysis was to read and get familiarised with the included studies. This was followed by creating a conceptual framework of categories on carers’ experiences as an emerging process using a few studies. Subsequent studies were coded into pre-existing concepts, and new categories were created when deemed necessary. As a third step, from these categories, themes of meaningful units are described and presented as findings. VS extracted and summarised the data for the results. MP and CJ reviewed and highlighted outstanding issues and final themes were subsequently arrived at through discussions. As this is an aggregative synthesis, we maintained reflexivity throughout the review process through discussions and reflections on extracted data and coding.

#### Question 1: identify the types and uses of AT in dementia

To date, there appears to be no agreed way of classifying AT available for use by people with dementia, and we have classified them by their use as part of this review. A list of AT described in the included studies (Table 2) was created with uses of the AT classified based on work developed by AT dementia [99] and Lorentz et al. [72]. From the included studies, AT is a mixture of active (requires action or interaction from the part of the person living with dementia or carer) and passive devices. Some devices had multiple uses e.g. the electronic medication reminders acted both to orient the person with dementia to time when they needed to take their medication as well as acting as a safety device to ensure they took important medication on time. 64 of the AT products described in the studies were commercially available with 10 studies describing AT that were research prototypes.

**Table 2:** Types and uses of Assistive Technology

Main Use	Type of Assistive Technology	Product Availability
Basic Activities of Daily Living	Assistive robot – ‘Ed’ - personal computer; microphones, LCD screen, speakers and web cameras [51]; Robotic assistants [76, 83]	Research prototype
Leisure and social interaction	Special remote control [58]/ Simple remote control to TV [48, 50, 64]	Commercial Product
	Wearable sensors [71]	Commercial Product
	Robotic Therapy ‘seal’ [67]	Commercial Product
	Talking cazette/magazine [58]	Commercial Product
	*Telephones:* Picture button telephone [46, 58, 75]/ Simple mobile phone [10, 48, 50, 69]/ Photo memory telephone [47]/Easy to use telephone [79]	Commercial Product
	Tablet computer [42, 70, 80] /MP3 player [89]	Commercial Product
Memory support	Memory aid [58, 88]/Memory games [87]/Information on village they live in [87]/Cognitive stimulation exercises [88]	Research prototype
	Memory Message [48, 57]	Commercial Product
	Timer to coffee machine [48, 50]	Research prototype
	*Locators:* Object locator [50]/ Lost item locator [35, 46, 75]/Locating technologies [81]	Commercial Product
Memory support and Orientation	Forget-Me-Not Calendar [57]	Commercial Product
	Message box connected to coffee machine [50]	Research prototype
	Information retrieval system [63]	Commercial Product
	Reminiscence tools [10, 69]	Research prototype
	*Clocks and calendars:* Electronic orientation clocks [10, 69]/ Night and Day Calendar [46, 48, 57, 75, 85]/ automatic day and date calendar [48]/automatic calendar [49, 50]/ Digital calendar with remote control [50]/ Memory clock [48, 50]/	Commercial Product
Memory support; Orientation; Safety and security	MeDose watch [57]	Commercial Product
	Integrated Rosetta system: Elderly Day Navigator + The early Detection System + Unattended autonomous surveillance system [90]	Research Prototype
	Assisted cognition system - touchscreen reminders for the day, current date and time, tracking [91]	Research Prototype
	Smart home management system (Xanboo) [38]/Smart home technologies [76]	Commercial Product
	Personalised recorded messages [72]	Commercial Product
Orientation; Safety and security	*Medicine reminders:* Electronic medication reminder [46, 47, 75, 88]; electronic pill dispensers [10, 35, 59, 69, 82]/ Medicine dispenser with alarm [48, 50]/Medication organiser [81]	Commercial Product
	COGKNOW day navigator - sensors and sensor network [65, 86]	Research prototype
	Talking motion sensors [72]	Commercial Product
Safety and security	Passive positioning alarm package [62, 73]	Commercial Product
	Safety alarm [58]	Commercial Product
	Bed alarm [58]/Bed occupancy sensor [81]	Commercial Product
	Door alarm [58, 79]	Commercial Product
	*Sensors:* Passage sensor [58]/Sensors [38, 39, 48, 63, 88]/Movement detectors [41, 47]/ Movement sensors [35, 81]/Proximity alarms [81]/Fall sensor [76]	Commercial Product
	*Global Positioning Systems:* Electronic tracking device [58]/ GPS [50, 59, 78, 92]/ GPS Location monitoring devices [10, 66, 69, 84]/Tracking devices [41, 48, 76]/GPS Tracking system [52, 54–56, 67, 74, 77]	Commercial Product/Research prototype
	Stove timer [50, 53]/ Cooker alarm [48]/Gas cooker device [46, 75]/ Automatic gas switch off devices [41]/Cooker monitors [58]	Commercial Product
	Electronic door lock [48, 50]/Touchpad key locks [81]	Commercial Product
	Talking wrist watch [48, 50]	Commercial Product
	ADLife - gateway with alarm button, sensors [61]	Research prototype
	In-home night time monitoring system [36, 37, 40]	Research prototype
	Automatic night lamp [46, 75]	Commercial Product
	Automatic water switch off devices [41]/Water detector alerts [81]	Commercial Product
	Baby monitors [68, 72]	Commercial Product
	Health monitoring system [67]	Commercial Product
	Emergency response system [39, 88]/Emergency transmitters [79]	Commercial Product
Safety and security, Social interaction	Skype (on computer/tablet PC) [68]; Social contact system [67]	Commercial Product
	NOCTURNAL - Sensors, communication devices, tablet PC [60]	Research prototype
	Cameras [35, 41, 47, 68, 72, 81]/ Video communication system [63, 76, 87, 88]/Video surveillance [76]	Commercial Product

The most commonly used AT was for safety and security (*n* = 38) including tracking devices and home safety devices. Followed by devices used for supporting memory and orientation for the person living with dementia (*n* = 23) and for social interaction and leisure activities (*n* = 16). In this review, very few studies (*n* = 3) considered AT which supported basic Activities of Daily Living activities such as feeding, washing, grooming or dressing. The AT used (including some research prototypes) are adapted from aids/devices that many people, with and without cognitive impairment, already use. None of the AT were for advanced instrumental Activities of Daily Living, such as managing finances, shopping or preparing meals and none of the AT addressed behavioural issues such as aggression or disinhibition, which is quite common in someone who has dementia.

#### Question 2: describe the effectiveness of AT for carers

The included studies reported on a wide range of carer-oriented measures (Zarit Burden interview, satisfaction with AT, carer well-being score), many of which were created for a specific study. A list of outcome measures used is presented in Additional file 3. Not all included studies reported on the effectiveness of AT for carers and due to the wide range of outcome measures and uses of AT, a descriptive summary of reported changes is provided (Table 3). From the 16 quantitative studies (17 publications), AT were reported as ‘somewhat’ or ‘very useful’ and AT is viewed as an adjunct to caregiving. There were no significant changes in carer reported well-being or burden. Surprisingly none of the studies considered or reported adverse events from AT use. Generally, carers reported they would recommend use of AT to others in similar situations, especially AT that supported safety and security for people with dementia. Where this was specifically asked, carers reported wanting to continue to use the AT, after the trial period. AT devices for safety, including tracking devices were the most used and appreciated by carers.

**Table 3:** Reported changes in informal carers

Studies	Positive change	Negative change	No change	Statistically significant change
Gitlin LN et al. [35]	Overall somewhat to very helpful.			
Rowe MA et al. [36]	• Experimental group 85% less likely to sustain an event. • Caregivers reported satisfaction and confidence in preventing night time injuries and exit using the NMS.			
Rowe MA et al. [37]			• No significant improvement in sleep for caregivers. • NMS not sufficient as standalone treatment.	
Olsson A et al. [73]	• Decreased level of worry about PwD’s independent outdoor activities.		• No significant changes in perceived well-being and burden.	
Pot AM et al. [74]	• Decrease in the feelings of worry when they could reach PwD. • 30% of carers reported they got time for other things since using the GPS.		• Feelings of role-overload were not significantly reduced during the study period.	
Kinney JM et al. [38]	• 87.5% of carers reported that the monitoring system made life easier (peace of mind, added security, easier to keep track of PwD). • 68.75% report that the system gave carers more free time and more time for self.	• 43.75% of carers report that the system made life more difficult (cell phone alerts can be annoying; one more thing to worry about)		
Duff P et al. [75] 2007	• Carer burden decreased very slightly during the course of the trial. • 100% of carers using picture telephone and cooker monitor reported satisfaction. • Over 75% of carers reported satisfaction with other AT used in the trial.			
Rialle V et al. [76]	• Tracking devices were better appreciated by women. • Younger caregivers found AT more useful than elderly.			
Landau R et al. [77]	• GPS device used for sake of patients’ safety or for carers’ peace of mind.			
Chen Y-C et al. [78]	• Most caregivers hope technological products (lost seeking devices) would increase the efficiency and safety			
Alwin J et al. [79]			• AT for time orientation, day planning and memory devices were more frequently associated with group of carers who reported some/no significant fulfilment and importance.	• Carers receiving alarm/security devices reported high fulfilment and importance.
Lim F S et al. [80]	• 47.63% of carers reported AT (iPad) was helpful			
McKenzie B et al. [81]	• AT devices provided immediate relief, reduce stress and helped carers provide care more easily and safely.			
Schulz R et al. [39]			• Caregivers balance costs against potential benefits such as improved functioning, increased autonomy, reduced burden, better health and enhanced safety.	
Kamimura T [82]	• Three caregivers maintained score of little burden or less and one caregiver had a score of mild burden throughout.			
Korchut A et al. [83]			• Reminders for medication was a high priority. • Carers viewed robotic technology positively.	
Topo P et al. [85] 2007	• 78% of carers found the night and day calendar useful 3 weeks after use and 82% after 6 months of use.			
Meiland F et al. [86] 2012			• No effect on burden or quality of life of the carers.	
Nijhof N et al. [87] 2013	• The cost analysis showed that it is more cost-effective for clients with dementia to live at home with the system [PAL4-dementia system] than to stay in a nursing home.			
Mehrabian S et al. [88] 2015	• 83% of carers felt the system [telecare prototype] had potential for helping in urgent situations. • 70% of carers felt that they would be ready and accept testing the system at home.			
Lewis V et al. [89] 2015	• 65% of carers comments were positive with respect to utility of the MP3 player.		• No change in self-rated general health. • No change in overall level of satisfaction	• Significant increase in the total Symptom Management Self-Efficacy score (a measure how confident the caregiver is that they will be able to manage problems that come up and deal with the frustrations of caring). Mean at baseline was 23.5 (SD = 6.1) and 27.0 after 4 weeks (SD = 7.5) (*t* = − 3.1, df = 47, *p* < 0.01).
Hattink B J et al. [90] 2016	• All informal carers felt the system [Rosetta] despite technical difficulties, is very useful and that they were happy with it.		• No significant differences on quality of life, perceived autonomy and feeling of competence between participants who used the Rosetta system and those who received usual care (the control group).	
Navarro R F et al. [91] 2016	• Caregiver burden levels show a decreasing trend, while levels of self-efficacy in caregivers increased by using the ambient assisted intervention system.			
Liu L et al. [92] 2017		• Some problems relate to false alarms and notifications.		
Tyack C et al. [42] 2017			• No significant change of quality of life or well-being across the intervention [tablet computer].	

#### Question 3: describe carers' experiences of AT use in dementia

Thematic synthesis from the qualitative data generated 4 themes and 15 sub-themes. Quotations from studies to support themes and sub-themes are listed in Table 4.

**Table 4:** Sample quotes for Themes and Sub-themes

Theme	Sub theme	Example Quotation 1	Example Quotation 2	Example Quotation 3
Positive aspects	Relationships	‘the use of the device generates longer instances of interaction’ [70].	‘Technology itself can become a ‘member’ of the social network, making it stronger’ [47].	‘I believe that my ability to have my mother continue to live with us would be dramatically reduced if we didn’t have NMS’ [40].
	Freedom and autonomy	‘As I say, *I couldn’t have continued working as long as I did*, and I’m still, we’re still benefitting from it [BUDDI device] you know. It, it’s really, I think it’s a wonderful device, wonderful’ [10].	‘In this way, the picture phone helped the person with dementia maintain independence, something the relatives described as important’ [58].	‘Informal caregivers, in contrast [to formal carers], request ICT solutions that enhance their personal freedom’ [63].
	Safety	‘I would like to keep it [the passive positioning alarm] ... // because it really provides security’ [62].	‘simple movement sensors or alarm systems that are networked to allow remote alerts were the most enthusiastically received’ [41].	‘Family caregivers expressed the belief that electronic tracking enables the patients’ independent outdoor mobility and at the same time improves their safety’ [55].
	Quality of life (stress, burden, wellbeing)	‘speaking watches" that read the time of day aloud at the push of a button. With this device, the caregivers experienced fewer questions and less stress and misunderstandings about the time’ [50].	‘The family caregivers were satisfied because the SRC [Simple Remote Control] removed both worries and burden of interruptions at work’ [64].	‘Informal caregivers reported that use of the system [preventative sensor technology] provided benefits to their mental well-being’ [61].
	Competence	‘it [GPS tracking device] was used to enable the person to continue to go out alone’ [56].	‘enabled them to better balance their needs for personal space with their desires to remain connected to the PWD during the night’ [40].	‘For the caregiver, data shows that the picture-button telephone was also most useful, with five out of six caregivers claiming they themselves were still using the product 3 months after its installation, and each reporting they considered it useful’ [46].
Negative aspects	Freedom and autonomy	‘Some participants feared that technology which simplified tasks too much might weaken a person’s own abilities such as in remembering numerical series and codes’ [59].	‘You can trust another person, but I think technology would be a bit … well, what if the technology went wrong? You can’t be 100% sure that the person would be cared for when you walk out of the door, can you? If somebody else is there, then you know’ [41].	‘Mrs B. pointed out the obligation to subscribe to an assistance platform. Mrs. B. would like to have the possibility to buy the device and manage by herself her husband’s wandering’ [54].
	Relationships	‘I think people need people – not just gadgets, you know? That’s the worrying thing really, with the elderly in particular. The gadgets replace people, and there isn’t any comparison’ [41].	‘The simple remote control used to be a great advantage for my wife, but now, after being at the hospital for some weeks, she doesn’t know how to operate it any longer. I have to tell her how to use it, all the time, and she is no longer able to use it when she’s alone’ [50].	‘We don’t want technology – we want people’ [41].
	Competence	‘Future willingness to use a technology generally outstripped their current willingness to use it’ [67].	‘As she was not competent in informatics, Mrs. B. had to rely on the personal care attendant: “She logs very easily and communicates information to me”. Thus, the situation was not under Mrs. B.’s control’ [54].	‘…..equipment may need some adjustments for use by elderly caregivers’ [52].
	Quality of life (Stress, burden, wellbeing)	'Adding activities into the [electronic] calendar was extremely time-consuming and complicated compared to an ordinary calendar: "I cannot sit here evening after evening and struggle with this computer!’ [50].	‘One of the caregivers reported that in some circumstances the system might increase the burden of care, if the sensors detected certain situations where additional care was required’ [61].	'…family members were not sure how to raise the issue of using an electronic tracking device: "How do you explain [to] your relatives that they will be monitored in all their outdoor activities?’ [55].
Use of AT	Ethical issues	‘The persons with dementia and their spouses saw the value of being locatable and saw no problem with the persons with dementia being monitored; they had not even considered that aspect’ [62].	‘Among the most central worries were fears of a dehumanized care’ [63].	‘…relatives shifted between their own needs for safety and security and what they perceived to be the need of the person with dementia when reflecting on the use of ICT’ [58].
	Help and support from carers	‘The engagement and interest of FC [Family Carers] was crucial in order to follow up the new AT device and support the person with YOD in using it’ [50].	‘Carers provide practical help that involves cognitive effort and is emotionally challenging’ [49].	‘the use of assistive technology was in some cases influenced by the availability of a caregiver willing to remind the person about the product’ [46].
	Raising issues of using AT	‘She said she felt, it (a pendant alarm) made her feel like a crock, you know (laughing). She says, “I don’t need this, I’m perfectly alright.” And the way that I persuaded her to wear it was, I said, “It just makes me feel better to know that you can contact somebody if you have a fall in the house, or if you’re not too well and you can’t get to the phone.” So, I said “You might not want to wear it, but wear it for me please because it, it stops me worrying about you.” Erm, so that was why she wore it, really’ [10].	‘The carers' attitude, commitment and will to learn about and follow through with the testing of the technology were vital if the equipment was to be useful and functional’ [48].	‘One participant had adjusted their newly purchased washing machine by labelling each compartment of the machine so that his wife would know where to put the washing detergent and the rinsing agent, thus enabling her to “still be ruler of the laundry room,” as he put it’ [59].
Acceptance and knowledge of AT	Costs and resources	‘It was striking that no participant talked about any time or money savings through using networked technologies’ [41].	‘Several carers noted that AT was generally expensive. You know, some people can’t afford it. I don’t mind paying for it ‘cause it’s helping her (mother) but I think it, it is expensive. It is quite steep, but then again, if her attendance money is there for it and she needs it, you, you don’t mind getting it if it’s going to help her, you know’ [10].	‘…when the participants considered technology to be beneficial to their relative with dementia or to themselves in their roles as significant others, they were ready to try technological solutions for support’ [59].
	Acceptance of AT	‘The participants saw technological innovations as an intrinsic feature of societal change and inevitable. The expectation was that the use of technology would increase, particularly for the next generation of carers who would have the aptitude and skills to adopt them’ [41].	‘One prerequisite for incorporation of technology emphasized in all groups was that technology must not be perceived as stigmatizing by the prospective user’ [59].	‘..the use and usefulness of the five products tested was largely determined by their technical capacity’ [46].
	Knowledge of Technology	‘Carers and GPs generally found the term AT unhelpful and open to interpretation…… “Well, I think the whole thing was introduced to me in a very nebulous way. Technology, what the hell does that mean?”’ [69].	‘Timely information is important for the FC, because the AT may become too complicated to handle for the person with YOD, as the dementia progresses’ [50].	‘Dementia caregivers' knowledge of new technologies lags behind current technology development’ [67].

#### Positive aspects

All the studies reported that the experience of cares using AT was generally positive.

##### Relationships

The use of AT for leisure and social interaction, memory support; orientation; safety and security seemed to help strengthen relationships between the person living with dementia and their carers. The AT was perceived as helping the carer function better in their caregiving role and became a ‘member’ of the wider social network of the person with dementia. For example, the use of a picture button telephone assisted a person with dementia in longer instances of interaction and maintaining social contacts with neighbours, friends and family.

##### Freedom and autonomy

Some of the studies reported carers having to use controlling methods such as locking and restricting access and the AT seemed to offer an alternative solution of enabling the person living with dementia to become independent and participate in meaningful activities. This in turn had a positive effect on the carers. The AT also provided carers with additional personal time which was highly valued and, in many instances, helped create the balance between their own personal space and independence with that of staying connected with the person with dementia.

##### Safety

Carers viewed someone who has dementia’s ability to stay in the community and their physical safety as more important than privacy and autonomy. Tracking devices that supported safety were enthusiastically received and AT provided carer reassurance and enhanced independence for both the carer and the person with dementia.

##### Quality of life

Whether the person living with dementia used the AT independently or the carer assisted them, AT was perceived as removing worries and burden and generally improved mental well-being, especially when the carer was living away from the person with dementia.

##### Competence

AT was perceived as improving independence for someone who has dementia, this had a positive effect on the carer, with some carers also reporting benefitting from using the AT themselves, such as the simple remote control for TV and memory aids.

#### Negative aspects

While the overall experience of AT use was perceived as positive by carers, some important negative aspects were also raised.

##### Relationships

When AT failed or the person living with dementia was no longer able to use the AT, this invariably caused constraints in the relationship, as an outcome of the presence of the AT. Some carers also perceived that the AT would replace the ‘person’ component of caring.

##### Freedom and autonomy

There were perceptions that the person living with dementia’s declining abilities could be further worsened using AT as they would no longer be actively challenged cognitively. Carers also believed that with the people with dementia who did not have adequate social care could be left alone with the technology without additional support for autonomy or social contact.

##### Competence

Carers seemed to be more willing to use AT in the future rather than currently. Elderly carers also worried about their competence and familiarity with AT, especially when there were technical failings with the AT or when the devices required to be replaced with new AT, as the illness progressed.

##### Quality of life

Occasionally, the use of AT seemed to create more dependence of the person with dementia on the carer, which led to increased stress for the carer, and the attitude of the person living with dementia towards the AT (from hostility to indifference) also led to additional carer burden, while choosing and using the AT.

#### Use of the AT

##### Ethical issues

Carers weighed the needs of personal reassurance and sense of security with that of autonomy of someone who has dementia while deciding on use of AT. Often there was no perceived ethical dilemma where the safety of the person with dementia was concerned. There was a consensus among carers that people with dementia must be involved as much as possible to select and use AT. Ethical issues around who held the power of choice of usage and discontinuance of AT and whether the needs of the person living with dementia were altered to match the potential of the currently available AT also seem to arise from the studies with no definitive conclusions.

##### Help and support from carers

Carers continuous engagement and willingness to provide support with the use of AT for the person with dementia was key in the use of AT in most of the studies. The carers’ attitude, commitment and willingness to learn about the AT were vital if the equipment was to be useful and functional.

##### Raising issues of using AT

Carers used different methods to convince people with dementia to accept and use AT, especially when the person living with dementia was hostile towards or did not understand the need to use the AT. Carers especially had difficulty convincing someone who has dementia where monitoring and safety devices were to be used compared to using AT for leisure and social interaction.

#### Acceptance and knowledge of AT

##### Costs and resource

Carers noted that AT was generally expensive, however most of the studies included in this review either provided the technology to the participants or participants did not mind spending the extra costs for AT that could support the person with dementia to stay for longer, in their own home.

##### Acceptance of AT

Many of the carers accepted AT as useful and their adoption depended on the perceived usefulness of the AT. They would also recommend its use to other carers and people with dementia. Carers also saw technological innovations as inevitable and expected the use of AT to increase and future generations of carers would have better skills and motivation to adopt them.

##### Knowledge of technology

There was a general feeling among carers that information regarding AT should be provided early in the process of diagnosis and support available to the person living with dementia, especially as the progress of dementia was unpredictable. The main need of information was on simple and practical AT solutions with most carers unaware of new AT devices and solutions available.

## Discussion

The aim of this systematic review is to identify the types and uses of assistive technology in dementia and describe the effectiveness and experience of its use for carers. The studies included cover the last 18 years and give a broad picture of AT use in dementia care. Caregiving for people with dementia in the community is usually unplanned, unpaid work carried out by the relative of the person living with dementia. The role of carer can be rewarding, but it can also be detrimental to a person’s well-being and can put them under a lot of stress [100, 101], especially for a carer who has little experience. AT is one way for supporting people with dementia and their carers to stay for longer in the community.

The symptoms which have the highest impact on carers of persons with dementia are repetitive questions, apathy, getting lost, aggression and incontinence [37, 40, 66] but the AT solutions from studies included in this review did not effectively address behavioural problems except safety/alert devices for wandering and getting lost. Fuhrer et al. [102] argue that effectiveness, efficiency, device satisfaction, psychological functioning and subjective wellbeing are essential outcomes for continued short-term and long-term use for AT. Findings from this review highlight that carers of people with dementia may prefer a specific type of AT, such as a GPS tracker, movement sensor or medication reminder and perceive it as useful but it may not have any real effect on outcomes of burden, satisfaction or wellbeing, similar to findings from other reviews on AT [27, 103]. One reason for this could be that existing outcome measures that are being used in AT studies may not be sensitive enough to measure change when using AT or are not valid in this context, perhaps as most measures were developed before AT was introduced.

This review highlights the continued lack of consistency in describing or classifying AT [104]. Other studies and reviews [7, 102, 105–107] have highlighted different ways of classifying AT used in dementia care. Having a classification system based on use (with more than one use per AT) from the perspective of the person with dementia and carer, as described in this review, may improve consistency of reporting and enhance synthesis of findings from trials and reviews. We have classified AT based on (i) Name of AT (ii) Type of AT (iii) function assisted (use or intended impact) and (iv) availability (commercial/prototype).

Though some research, involving robotic technology in institutional and simulation/lab based settings is looking into this [108, 109], this review identified the lack of sufficient number of AT to support basic and instrumental Activities of Daily Living for people living with dementia at home. This could be because it is difficult to develop and deploy potentially bulky/expensive AT in a non-institutional setting or perhaps human/assisted care is seen as easier and less expensive way of providing this care [110]. It is also possible that technological advances in miniaturisation and artificial intelligence have not yet caught up with this area of need.

It is also clear from this review that installation of AT at home for use by someone who has dementia was often wrongly seen as a one-off event, rather than an ongoing process for getting the best out of AT. Similar to other findings [111–114], this review found that carers as users of AT often struggle to understand and engage with the technology in their homes as a result of poor understanding, a lack of knowledge of available AT and lack of on-going support from professionals and design flaws in the AT itself.

The review also highlights the perceived fear among some carers that use of AT could lead to social isolation. However available AT solutions such as tablet computers and monitoring devices to alert carers gives them a sense of participating in the life of a person living with dementia even when the carer is not physically present, this led to AT being viewed as a positive addition. There was no evidence within the included studies that multiple AT solutions were being harnessed to bring them together for an integrated solution that could assist both people with dementia and carers. AT devices were used in isolation for specific functions rather than a combined use of the devices. With the rise of internet of things [115, 116] and connected AT devices combining multiple AT for use with a person with dementia or carer is feasible and in most instances more desirable [117].

Interestingly all the studies considered the introduction of AT after a diagnosis of dementia, the timing of introducing devices may be important. Safety/tracking devices were introduced pre-emptively to prevent secondary problems [7, 27] such as falls and wandering, which in turn could potentially reduce admissions into long-term care [118] but equal consideration and further research may be needed for the use of AT as a preventative measure especially in areas of orientation, memory and leisure.

Many of the installed AT did not meet the needs of the user. Despite a surprising lack of reporting on adverse events, some of the negative reactions to AT were because they were ‘Off the shelf’ devices and were rarely useful, especially with a progressive condition like dementia. The AT needed to be adapted or customised for the carers and people with dementia’s individual needs and when this was not the case, led to abandonment of the AT [117, 119, 120]. Co-creating AT with users has steadily improved over time. Carers need to be involved in the design and testing of AT solutions and in prioritising the problems that need to be addressed to allow AT to be accepted as a solution for caring for people living with dementia in the community [121, 122].

## Implications and recommendations from this review

1. The function assisted domain (e.g. Memory device, GPS tracker) as a way of naming the AT is usually defined by the manufacturer/developer of the AT. We recommend a shift towards considering naming the use of the AT from the perspective of the person with dementia and their carer to ensure that device is appropriately used and can provide the intended benefits of that AT [123] for both the carer and the person living with dementia.
2. Further research should be carried out on how multiple AT devices could work together or be combined to better support someone who has dementia and their carers rather than how individual AT devices can support them.
3. Future research should focus on AT solutions which are co-designed by those with lived experience of the challenges of dementia at home and should include carers, who live with and away from a person with dementia.
4. Ability of a carer to ‘problem solve’ should be a consideration in AT prescription and use. Technology should match the needs of the person requiring the use of the AT, rather than the person being ‘moulded’ to match what technology is available for them.

## Limitations

Due to the variety of AT devices and outcome measures used, we could not pool results from the quantitative studies and have provided a narrative review instead. Due to financial constraints we did not include studies in languages other than English within this review and this could have potentially led to some suitable studies being missed. However, we did scan for reference lists of all studies that were included for full text review and are confident that this review captures all suitable studies that met our inclusion criteria.

## Conclusions

Technology is advancing at an extremely rapid pace, especially within the fields of artificial intelligence and machine learning with their resultant healthcare applications. It is likely that AT powered by AI may become ubiquitous soon. The quality of research focussing on AT use in dementia continues to be low. AT solutions helps improve carers’ experience of providing care to a person living with dementia. AT would support people with dementia and carers in the community but researchers, healthcare professionals and technology developers should adopt a family centred model for use of AT than pursuing only an individual/person centred model of care.

## Additional files

Additional file 1: Search strategy. (DOCX 18 kb)

Additional file 2: Data extraction forms. (DOCX 15 kb)

Additional file 3: Data from included studies. (DOCX 114 kb)

Additional file 4: PRISMA checklist. (DOCX 27 kb)

## Data Availability

All data generated or analysed during this study are included in this published article [and its supplementary information files].

## Abbreviations

ALOIS: ALOIS, named after Alois Alzheimer, is a register of dementia studies maintained by the Cochrane Dementia and Cognitive Improvement Group
AMED: Allied and Complementary Medicine Database
AT: Assistive technology
CINAHL: Cumulative Index of Nursing and Allied Health Literature
PRISMA: Preferred Reporting Items for Systematic Reviews and Meta-Analyses
PROSPERO: International Prospective Register of Systematic Review
