# Supplementary material for: Informal carers’ experience of assistive technology use in dementia care at home: a systematic review
Source: BMC Geriatr. 2019 Jun 14;19:160. doi: 10.1186/s12877-019-1169-0 (PMC6567448; doi:10.1186/s12877-019-1169-0)
Supplement: Supplementary file 1 — Search strategy. (DOCX 18 kb) [file 12877_2019_1169_MOESM1_ESM.docx]

**Additional file 1: Search strategy**

Database: Ovid MEDLINE(R) Epub Ahead of Print, In-Process & Other Non-Indexed Citations, Ovid MEDLINE(R) Daily and Ovid MEDLINE(R) <1946 to Present>

Search Strategy:

# ▲ Searches

1 exp Dementia/ or dement*.mp. (197638)

2 (lewy bodies or alzheimer*).mp. (150642)

3 exp Wernicke Encephalopathy/ or wernicke encephalopath*.mp. (1915)

4 exp Korsakoff Syndrome/ or korsakoff.mp. (1214)

5 1 or 2 or 3 or 4 (243505)

6 Technology/ or technolo*.mp. (468577)

7 (informat* adj2 communicat*).mp. (7204)

8 ict.mp. (45500

9 Information Systems/ or Telemedicine/ or Monitoring, Ambulatory/ (45266)

10 (telemedicine or tele medicine or telehealth or tele health or telemonitor* or tele monitor* or ehealth or e-health or mhealth or m-health).mp. (28210)

11 Telephone/ or remote consultation/ (16139)

12 "Activities of Daily Living"/ or Self-Help Devices/ or assistive.mp. (70880)

13 device*.mp. (417837)

14 self care.mp. or Self Care/ (41709)

15 self help.mp. (18226)

16 (gero* adj2 tech*).mp. (35)

17 robo*.mp. or Robotics/ (41477)

18 (assist* adj2 (aid? or device?)).mp. (20630)

19 "Equipment and Supplies"/ or Equipment Design/ or equipm*.mp. (314337)

20 Signal Processing, Computer-Assisted/ or Clothing/ or wearable*.mp. (61224)

21 6 or 7 or 8 or 9 or 10 or 11 or 12 or 13 or 14 or 15 or 16 or 17 or 18 or 19 or 20 (1312200)

22 Caregivers/ or caregiv*.mp. (68731)

23 (relatives or family or families).mp. or exp Family/ (1264961)

24 friend*.mp. or Friends/ (74209)

25 (neighbour? or neighbor?).mp. (27191)

26 Spouses/ or spous*.mp. (30883)

27 (wife or wives).mp. (10105)

28 husband?.mp. (11790)

29 (couple? or partner?).mp. (463702)

30 son?.mp. (46342)

31 daughter?.mp. (25773)

32 Home Nursing/ or carer*.mp. (21540)

33 Home Care Services/ or home.mp. (227871)

34 22 or 23 or 24 or 25 or 26 or 27 or 28 or 29 or 30 or 31 or 32 or 33 (2014136)

35 5 and 21 and 34 (3857)

Database: Embase <1974 to 2017 December 15>

Search Strategy:

1 exp Dementia/ or dement*.mp. (327618)

2 (lewy bodies or alzheimer*).mp. (207698)

3 exp Wernicke Encephalopathy/ or wernicke encephalopath*.mp. (2641)

4 exp Korsakoff Syndrome/ or korsakoff.mp. (2461)

5 1 or 2 or 3 or 4 (359221)

6 Technology/ or technolo*.mp. (576145)

7 (informat* adj2 communicat*).mp. (8326)

8 ict.mp. (6519)

9 Information Systems/ or Telemedicine/ or Monitoring, Ambulatory/ (62971)

10 (telemedicine or tele medicine or telehealth or tele health or telemonitor* or tele monitor* or ehealth or e-health or mhealth or m-health).mp. (31229)

11 telephone*.mp. or exp Telemedicine/ or exp Online Therapy/ (102636)

12 "Activities of Daily Living"/ or Self-Help Devices/ or assistive.mp. (61648)

13 device*.mp. (618224)

14 self care.mp. or Self Care/ (51841)

15 self help.mp. (16621)

16 (gero* adj2 tech*).mp. (33)

17 robo*.mp. or Robotics/ (59589)

18 (assist* adj2 (aid? or device?)).mp. (31835)

19 "Equipment and Supplies"/ or Equipment Design/ or equipm*.mp. (402108)

20 Signal Processing, Computer-Assisted/ or Clothing/ or wearable*.mp. (51185)

21 assistive technology.mp. or exp Assistive Technology/ (3349)

22 6 or 7 or 8 or 9 or 10 or 11 or 12 or 13 or 14 or 15 or 16 or 17 or 18 or 19 or 20 or 21 (1714806)

23 Caregivers/ or caregiv*.mp. (90598)

24 (relatives or family or families).mp. or exp Family/ (1426264)

25 friend*.mp. or Friends/ (86423)

26 (neighbour? or neighbor?).mp. (26420)

27 Spouses/ or spous*.mp. (26242)

28 (wife or wives).mp. (11096)

29 husband?.mp. (12779)

30 (couple? or partner?).mp. (498454)

31 son?.mp. (147451)

32 daughter?.mp. (29845)

33 Home Nursing/ or carer*.mp. (70884)

34 Home Care Services/ or home.mp. (321048)

35 exp Home Care/ or exp Family Members/ or informal carer.mp. (65774)

36 23 or 24 or 25 or 26 or 27 or 28 or 29 or 30 or 31 or 32 or 33 or 34 or 35 (2392642)

37 5 and 22 and 36 (5646)

Database: PsycINFO <1806 to December Week 2 2017>

Search Strategy:

1 exp Dementia/ or dement*.mp. (86717)

2 (lewy bodies or alzheimer*).mp. (56910)

3 exp Wernicke Encephalopathy/ or wernicke encephalopath*.mp. (68)

4 exp Korsakoff Syndrome/ or korsakoff.mp. (715)

5 1 or 2 or 3 or 4 (94803)

6 Technology/ or technolo*.mp. (102473)

7 (informat* adj2 communicat*).mp. (7270)

8 ict.mp. (3106)

9 Information Systems/ or Telemedicine/ or Monitoring, Ambulatory/ (9321)

10 (telemedicine or tele medicine or telehealth or tele health or telemonitor* or tele monitor* or ehealth or e-health or mhealth or m-health).mp. (5946)

11 telephone*.mp. or exp Telemedicine/ or exp Online Therapy/ (29363)

12 "Activities of Daily Living"/ or Self-Help Devices/ or assistive.mp. (8598)

13 device*.mp. (36483)

14 self care.mp. or Self Care/ (10675)

15 self help.mp. (9405)

16 (gero* adj2 tech*).mp. (24)

17 robo*.mp. or Robotics/ (7381)

18 (assist* adj2 (aid? or device?)).mp. (1214)

19 "Equipment and Supplies"/ or Equipment Design/ or equipm*.mp. (10071)

20 Signal Processing, Computer-Assisted/ or Clothing/ or wearable*.mp. (2639)

21 assistive technology.mp. or exp Assistive Technology/ (2236)

22 6 or 7 or 8 or 9 or 10 or 11 or 12 or 13 or 14 or 15 or 16 or 17 or 18 or 19 or 20 or 21 (203973)

23 Caregivers/ or caregiv*.mp. (52683)

24 (relatives or family or families).mp. or exp Family/ (367818)

25 friend*.mp. or Friends/ (62690)

26 (neighbour? or neighbor?).mp. (6767)

27 Spouses/ or spous*.mp. (26060)

28 (wife or wives).mp. (14604)

29 husband?.mp. (12939)

30 (couple? or partner?).mp. (121683)

31 son?.mp. (23554)

32 daughter?.mp. (12756)

33 Home Nursing/ or carer*.mp. (8884)

34 Home Care Services/ or home.mp. (107148)

35 exp Home Care/ or exp Family Members/ or informal carer.mp. (140630)

36 23 or 24 or 25 or 26 or 27 or 28 or 29 or 30 or 31 or 32 or 33 or 34 or 35 (679293)

37 5 and 22 and 36 (1653)

Database – CINAHL, Interface - EBSCOhost Research Databases 
Search Strategy:

S1 (MH "Frontotemporal Dementia+") OR (MH "Dementia, Vascular+") OR (MH "Delirium, Dementia, Amnestic, Cognitive Disorders+") OR (MH "Dementia, Multi-Infarct") OR (MH "AIDS Dementia Complex") OR (MH "Lewy Body Disease") OR (MH "Dementia, Senile+") OR (MH "Dementia, Presenile+") OR (MH "Kohlschutter-Tonz Syndrome") OR (MH "Dementia") OR "Dementia/ or dement*.mp." (58,106)

S2 (MM "Wernicke's Encephalopathy") OR (MM "Korsakoff Syndrome") (145)

S3 (S1 OR S2) (58,204)

S4 (MM "Assistive Technology Services") OR (MM "Technology+") OR (MM "Assistive Technology") OR "Technology/ or technolo*.mp." (17,508)

S5 "ict.mp." (0)

S6 (MM "Home Health Care Information Systems") OR "Information Systems/ or Telemedicine/ or Monitoring, Ambulatory/" (82)

S7 (MH "Telehealth+") OR "(telemedicine or tele medicine or telehealth or tele health or telemonitor* or tele monitor* or ehealth or e-health or mhealth or m-health).mp." (10,199)

S8 "telecare" (257)

S9 (MM "Remote Consultation") OR (MM "Telephone Consultation (Iowa NIC)") OR (MM "Telephone+") (3,485)

S10 (MH "Self-Care: Instrumental Activities of Daily Living (Iowa NOC)") OR (MH "Self Care: Activities of Daily Living (Iowa NOC)") OR (MH "Activities of Daily Living+") OR (MH "Assistive Technology Devices+") OR (MH "Instrumental Activities of Daily Living Alteration (Saba CCC)") OR (MH "Instrumental Activities of Daily Living (Saba CCC)") OR (MH "Activities of Daily Living Alteration (Saba CCC)") OR (MH "Activities of Daily Living (Saba CCC)") OR (MH "Altered Activities of Daily Living (NANDA)+") OR ""Activities of Daily Living"/ or Self-Help Devices/ or assistive.mp." (57,900)

S11 (MM "Assistive Technology") OR "assistive technology" OR (MM "Assistive Technology Services") OR (MM "Assistive Technology Devices+") (18,632)

S12 (MH "Toileting Self Care Deficit (NANDA)") OR (MH "Self-Care Deficit (Saba CCC)") OR (MH "Self-Care Component (Saba CCC)") OR (MH "Self-Care Assistance: Dressing-Grooming (Iowa NIC)") OR (MH "Self-Care Assistance: Bathing-Hygiene (Iowa NIC)") OR (MH "Feeding Self Care Deficit (NANDA)") OR (MH "Dressing-Grooming Self Care Deficit (NANDA)") OR (MM "Self Care+") OR (MH "Bathing-Hygiene Self Care Deficit (NANDA)") OR (MH "Self-Care: Toileting (Iowa NOC)") OR (MH "Self-Care: Oral Hygiene (Iowa NOC)") OR "self care.mp. or Self Care/" (16,363)

S13 (MM "Robotics+") OR "robo*.mp. or Robotics/" (2,287)

S14 (MH "Equipment and Supplies") OR (MH "Equipment Design") OR (MH "Home Care Equipment and Supplies") OR ""Equipment and Supplies"/ or Equipment Design/ or equipm*.mp." (32,718)

S15 (MM "Signal Processing, Computer Assisted") OR (MH "Therapy, Computer Assisted+") OR (MH "Computers, Hand-Held+") OR (MH "Computer Aided Design+") OR "Signal Processing, Computer-Assisted/ or Clothing/ or wearable*.mp." (9,942)

S16 S4 OR S5 OR S6 OR S7 OR S8 OR S9 OR S10 OR S11 OR S12 OR S13 OR S14 OR S15) (139,248)

S17 (MH "Caregiver Burden") OR (MH "Risk for Caregiver Role Strain (NANDA)") OR (MM "Caregivers") OR (MM "Caregiver Support") OR (MH "Family Caregiver Status (Iowa NOC)+") OR (MH "Caregiver Well-Being (Iowa NOC)") OR (MH "Caregiving Endurance Potential (Iowa NOC)") OR (MH "Caregiver-Patient Relationship (Iowa NOC)") OR (MH "Caregiver Support (Iowa NIC)") OR (MH "Caregiver Stressors (Iowa NOC)") OR (MH "Caregiver Strain Index") OR (MH "Caregiver Role Strain (NANDA)") OR (MH "Caregiver Physical Health (Iowa NOC)") OR (MH "Caregiver Performance: Direct Care (Iowa NOC)") OR "Caregivers/ or caregiv*.mp." OR (MH "Caregiver Role Strain (Saba CCC)") (18,827)

S18 (MH "Extended Family") OR (MH "Family Relations") OR (MH "Dependent Families") OR (MH "Patient-Family Relations") OR "(relatives or family or families).mp. or Family/" (12,213)

S19 (MH "Support System Enhancement (Iowa NIC)") OR (MH "Social Networks") OR (MH "Home Nursing") OR "friend*.mp. or Friends/" (12,236)

S20 "(neighbour? or neighbor?).mp." (1)

S21 (MH "Spouses") OR (MH "Significant Other") OR (MH "Caregivers") OR "Spouses/ or spous*.mp." (26,686)

S22 (MH "Spouses") OR "(wife or wives).mp." (6,262)

S23 "husband*.mp." (0)

S24 "husband*.mp." (2)

S25 "(couple* or partner*).mp." (0)

S26 "(couple* or partner*).mp." (531)

S27 "son*.mp." (0)

S28 "son*.mp." (1)

S29 (MH "Daughters") OR "daughter*.mp." (985)

S30 (MH "Home Nursing") OR "Home Nursing/ or carer*.mp." (2,836)

S31 (MH "Home Health Care") OR (MH "Home Care Equipment and Supplies") OR (MH "Home Health Care Information Systems") OR "Home Care Services/ or home.mp." (16,568)

S32 (S17 OR S18 OR S19 OR S20 OR S21 OR S22 OR S23 OR S24 OR S25 OR S26 OR S27 OR S28 OR S29 OR S30 OR S31) (66,762)

S33 S3 AND S16 AND S32 (810)
