# Supplementary material for: Informal carers’ experience of assistive technology use in dementia care at home: a systematic review
Source: BMC Geriatr. 2019 Jun 14;19:160. doi: 10.1186/s12877-019-1169-0 (PMC6567448; doi:10.1186/s12877-019-1169-0)
Supplement: Supplementary file 3 — Data from included studies. (DOCX 114 kb) [file 12877_2019_1169_MOESM3_ESM.docx]

**Additional file 3:** Data from included studies

| Qualitative studies | | | | | | | | | | |
| --- | --- | --- | --- | --- | --- | --- | --- | --- | --- | --- |
| No | Authors  Date | Country | Participants | Age range | Study Design | Theoretical Framework | Assistive Technology | Mixed Methods Appraisal Tool (%) | Data collected from | Major Findings |
| 1 | Altus DE et al^1^  2000 | USA | 2- Spouses  (2 men) | Not reported | Interviews | Case study | GPS tracker/Mobile locator | 50  (Findings within context and researcher influence on results unclear) | Participant reflections and diary of incidents | AT use provided increased confidence and peace of mind |
| 2 | Cahill S^2^  2007 | Ireland | 20 – Spouses + Children + Sibling + Daughter-in-law + Friend  (5 men; 15 women) | 34-79 | Semi-structured interviews | Thematic Analysis | Automatic night and day calendar; lost item locator; automatic night lamp; gas cooker device; picture button telephone; | 100 | Participant reflections | Low technical difficulty had highest use; caregiver willingness to remind PwD to use the AT enabled better use; Familiarity of AT to an older PwD influenced use. |
| 3 | Starkhammar S et all^3^  2008 | Sweden | 14 Spouses + Daughters  (5 men; 9 women) | Not reported | Interviews and Observations | Grounded Theory | Stove timer | 50  (Findings within context and researcher influence on results unclear) | Participant reflections | Safety issues were of concern for carers |
| 4 | Faucounau V et al^4^  2009 | France | 1 – Spouse  (1 woman) | 68 | Interviews | Single dyad case study | GPS tracker | 75  (Researchers’ influence on results unclear) | Participant reflections | Aesthetic aspects of the device; knowing how to use the internet; dissensions between patient and carer on carrying the device all influenced use of AT |
| 5 | Spring HJ^5^  2009 | USA | 14 – Spouses + Daughters + Grand daughter  (1 men; 13 women)  Ethnicity – White, Hispanic, African American | 38-86 | Semi-structured interviews | Full conceptual description- grounded theory | Inhome night time monitoring system | 75  (Consideration of researchers’ influence on results unclear) | Participant reflections | Peace of mind. Some increase in caregiver worry; balance between personal space and remain connected with PwD; worry decreased with trust in the AT; better sleep; energy for self-care and care for PwD. ? Financial benefits and positive impact on other family members |
| 6 | Landau R et al^6^  2010 | Israel | 36 – Spouses + Children  (12 men; 24 women) | Not reported | Focus groups | Thematic framework approach | GPS electronic tracking device | 50  (Process for analysing data unclear and researchers’ influence on results unclear) | Participant reflections on vignettes | Need for AT at different phases of the disease; safety and peace of mind; use after first time getting lost; staying in the community and safety more important than patient autonomy; get patient consent; |
| 7 | Powell J et al^7^  2010 | England | 34 – Spouses + Children + Grandchildren + Friend  (12 men; 22 women)  Ethnicity – White British, Other | 23-91 | Semi-structured interviews | Framework approach | Movement sensors; cameras; automatic water and gas switch off devices; tracking devices | 50  (Findings within context and researcher influence on results unclear) | Participant reflections on vignettes | Personal nature of human contact versus impersonal nature of technology; AT for others than themselves; useful for next generation of carers; trade-off between privacy, autonomy and safety; low tech solutions as important as advanced technology |
| 8 | White EB et al^8^  2010 | England | 10 – Spouses + Son+ Daughter  (4 men; 6 women) | 44-73 | Interviews and survey | Grounded Theory | GPS tracker | 75  (Researchers’ influence on results not explained) | Participant reflections and Machin carer strain scale (modified) | Enable people to participate in meaningful activities; tracking as a secondary intervention; reassurance and enhanced independence |
| 9 | Rosenberg L et al^9^  2011 | Sweden | 4 – Son +Daughters +Neighbour  (1male; 3 women) | 55-78 | Observations and In-Depth interviews | Grounded Theory | Night and Day Calendar, Forget-Me-Not Calendar, Memory Message, MeDose watch | 75  (Researchers’ influence on results unclear) | Participant reflections | Issues relating to who has power to decide on AT |
| 10 | Olsson A et al^10^  2012 | Sweden | 14 Spouses  (6men; 8 women) | 62-89 | Interviews | Not reported | Safety alarm, bed alarm, door alarm, passage sensor, electronic tracking device, cooker monitors, talking cazette, picture button telephone, memory aid, special remote control | 50  (Theoretical framework used is not reported and researcher influence on results unclear) | Participant reflections | Important to let PwD be involved in decisions about ICT use |
| 11 | Rosenberg L et al^11^  2012 | Sweden | 16 – Spouses + Son +Daughter+ Neighbour  (5 men; 11 women) | 45-78 | Interviews and Focus groups | Grounded Theory | GPS monitor, electronic pill dispenser | 75  (Researchers’ influence on results unclear) | Participant reflections | Safety issues overshadowed ethical dilemmas |
| 12 | Martin S et al^12^  2013 | Ireland | 8  (Gender not reported separately) | Not reported | Semi-structured interviews | Phenomenological approach | NOCTURNAL - Sensors, communication devices, tablet PC | 75  (Researchers’ influence on results unclear) | Participant reflections | High anxiety and increased time spent with PwD; gaining ethical approval when tech is in design phase is difficult |
| 13 | Nijhof N et al^13^  2013 | Netherlands | 14 – Spouses + Family + Friends  (Gender not reported) | Not reported | Semi-structured interviews | Not reported | ADLife - gateway with alarm button, sensors | 50  (Theoretical framework for analysing data not reported and researchers’ influence on results unclear) | Participant reflections | Study reports ethics was not required; anxiety from informal carers about real time video images; problems with equipment; useful if dementia deteriorates |
| 14 | Olsson A et al^14^  2013 | Sweden | 5 Spouses (2men; 3 women) | 62-68 | Participant Observation | Ethnographic approach | Passive Positioning Alarm | 50  (Findings within context and researcher influence on results unclear) | Participant experience | Being visible outweighs risk of privacy violated |
| 15 | Riikonen M et al^15^  2013 | Finland | 25 – Spouses + Daughters + Brothers + Son-in-law + Niece  (12 men; 13 women) | Less than 65 | Unstructured interviews and participant observations | Thought entity | Cameras, movement detectors, electronic medication reminder, photo memory telephone | 100 | Participant reflections | technology itself can become a member of the social network making it stronger; crisis following diagnosis complicates introduction of technology |
| 16 | Hastall MR et al^16^  2014 | Germany | 17 - Spouses + Children  (4 men; 13 women) | 38-91 | Semi-structured interviews | Thematic Analysis | Information retrieval system; Video communication system; sensors | 50  (Findings within context and researcher influence on results unclear) | Participant reflections on vignettes | Fears of dehumanized care; end user lack of experience with technology; informal carers preferred trustworthy carer related information in comparison to leisure time support |
| 17 | Jentoft R et al^17^  2014 | Norway | 9 – Son + Spouses +Daughters +Mother  (4 men; 5 women) | 19-89 | In-depth interviews and Observations | Social situated learning | Simple remote control for TV | 75  (Researchers’ influence on results unclear) | Participant reflections | AT helped PwD and Carer experience better quality of time when together |
| 18 | Meiland F et al^18^  2014 | Netherlands and Germany | 13  (Gender not reported separately) | Not reported | Interviews and Focus group | Not reported | COGKNOW day navigator; sensors and sensor network | 50  (Theoretical framework for analysing data not reported and researchers’ influence on results unclear) | Participant reflections on development of AT + Vignette | Carers preferred support from AT for navigation outdoors and calendar function; learning of new equipment raised as an issue; |
| 19 | Milne H et al^19^  2014 | Scotland | 16 - Spouses + Sister + Son + Daughters + Son-in-law + Daughter-in-law  (7men; 9 women) | Not reported | Interviews  (part of observational mixed methods study) | Thematic analysis with constant comparison | GPS devices - worn as watches, pendants or carried in pockets and bags | 75  (Researchers’ influence on results unclear) | Participant reflections | Still used locked door as trust of device was low; false sense of security from use of devices; Equipment was bulky; false alerts also occurred which worried carers. |
| 20 | Burstein AA et al^20^  2015 | USA | 34 - Spouses + Children + Grandchildren + Friend + Neighbour + Sibling + Niece + Daughter-in-law + Cousin  (6 men; 28 women) | 43-76 | In-depth interviews | Not reported | Sensor technology, GPS tracking device. Plus emerging technology - robotic therapy seal, GPS tracking system; social contact system, health monitoring system | 50  (Theoretical framework for analysing data not reported and researchers’ influence on results unclear) | Participant reflections on awareness of technology | Carers knowledge lags behind current technology development; Need for better education and raise awareness among carers; Future willingness outstripped current willingness to use AT; Need to alert carers of options for different stages of the disease. |
| 21 | Gibson G et al^21^  2015 | England | 26 - Spouses and Daughters  (Gender not reported separately) | 49-82 | Interviews | Thematic analysis, constant comparative method | Community alarms and telecare; GPS location monitoring devices, signage, reminiscence tools, clocks to aid orientation, simplified telephones with pictures, pill dispensers | 75  (Researchers’ influence on results unclear) | Participant reflections | AT accepted by PwD as it helped carer |
| 22 | Arntzen C et al^22^  2016 | Norway | 14 - Spouses +Children +Parent  Gender not reported) | 19-89 | In-depth interviews and Observations | Phenomenological conception of 'lived body' | Sensors, timers, tracking device, cooker alarm, timer on coffee machine, automatic day and night calendar, simple remote control for television, electronic door lock, item locator, simple mobile phone, automatic day and date calendar, speaking arm-wrist watch, message box reading out a message when activated, memory clock, medicine dispenser with alarm | 100 | Participant reflections | AT had to interest and engage family carers |
| 23 | Grigorovich A et al^23^  2016 | Canada | 20 – Sons + Son-in-law  (20 men) | 25-66 | Semi-structured interviews | Naturalistic enquiry, qualitative descriptive design | Cameras, baby monitors, skype | 75  (Researchers’ influence on results unclear) | Participant reflections | Sons relied on other caregivers for support. AT used mainly for monitoring and reassurance of safety; extension of established familial roles |
| 24 | Newton L et al^24^  2016 | England | 26  (Gender not reported separately) | 49-82 | Interviews | Not reported | Community alarms and telecare; GPS location monitoring devices, signage, reminiscence tools, clocks to aid orientation, simplified telephones with pictures, pill dispensers | 75  (Researchers’ influence on results unclear) | Participant reflections | AT useful and accepted as long as it helped PwD |
| 25 | Ekstrom A et al^25^  2017 | Sweden | 1 – Spouse  (1 male) | Not reported | Video recording and observation | Talk-in interaction | Tablet computer | 50  (Findings within context and researcher influence on results unclear) | Length and domains of conversation | Carers concern is on how is supports PwD |
| 26 | Evans N et al^26^  2017 | England | 6 – Daughters +Spouses +Daughter-in-law  (6 women) | 48-62 | Interviews and Focus groups | Interpretative Phenomenological Analysis | Calendar | 100 | Participant reflections | Little expectation from AT - does it work? |
| 27 | Hassan L et al^27^  2017 | England | 4  (Gender not reported) | Greater than 65 | Focus group | Not reported | Wearable sensors | 25  (Theoretical framework for analysing data not reported, results not explained in context and researchers’ influence on results unclear) | Participant reflections and discussion on vignettes | Did not get ethics approval; Carers expected researchers to intervene if devices picked up signs of treatable health problems; prefer low maintenance and robust devices; carers deeply involved in everyday routines of PwD; not to stereotype that older users cannot cope with technology |
| 28 | Holthe T et al^28^  2017 | Norway | 13 – Spouses + Daughter + Mother + son  (7 men; 6 women) | 19-89 | Interviews | Epistemology of coping | stove timer, timer to coffee machine, GPS, automatic calendar, simple remote control to TV, Electronic door lock, object locator, simple mobile phone, digital calendar with remote control, talking wrist watch, medicine dispenser with alarm, memory clock, message box connected to coffee machine | 100 | Participant reflections | "window" for successful implementation of AT may be short |
| 29 | Lorenz K et al^29^  2017 | England | 7 – Sons + other carers  (Gender not reported) | Not reported | Email and blog reviews | Not reported | Baby monitors, talking motion sensors, personalised recorded messages, cameras | 50  (Theoretical framework for analysing data not reported and researchers’ influence on results unclear) | Reports on personal evaluation of technology | Rapid literature review + emails and blogs; technology helps coordinate care between different family members; not many devices to support severe dementia. |
| 30 | Wang RH et al^30^  2017 | Canada | 10 – Spouses + Son + Daughters  (4 men; 6 women) | 36-78 | Semi-structured interviews | Thematic analysis | Assistive robot - Ed - personal computer; microphones, LCD screen, speakers and web cameras | 100 | Participant reflections based on observations | Robots - thought provoking and novel; did not need at present (psychological readiness) but future possibility; carers felt (incorrectly) PwD were as open as they in using and integrating robots at home; useful to reduce anxiety and answer repetitive questions; robot as an appliance rather than a friend |

| Quantitative studies | | | | | | | | | | |
| --- | --- | --- | --- | --- | --- | --- | --- | --- | --- | --- |
| No | Authors  Date | Country | Participants  Ethnicity (where reported) | Age range | Study Design | Assistive Technology | MMAT Appraisal (%) | Measures | Validated measures | Major Findings |
| 31 | Gitlin LN et al^31^  2010 | USA | 63/73 [Experimental/Control] – Spouse + Others  (13 Men; 50 women – Experimental group)  Dropouts not reported  Ethnicity – White, African American, Other | 53-79 | Randomised controlled trial | Medication dispensers, video cameras, motion detectors, lost item finders | 20  (No information on randomisation, no information on attrition and blinding) | 1. Use of device (yes/no).  2. Extent to which helpful (1=not helpful, 2=somewhat helpful, 3=very helpful) | No | - 87.6% of devices in use at 4months - Overall somewhat to very helpful. - Lower cognitive status equated to more AT. - Devices assisted caregivers in helping to carry out specific tasks or to minimise targeted problem behaviour. - AT provided only after caregiver approval. - Device use alone may not sufficiently address problem behaviours. |
| 32 | Rowe MA et al ^32^  2009 | USA | 26/27 [Experimental/control] – Spouses + Daughter + Son + Granddaughter  (7/4 men; 19/23 women).  Dropouts = 10 in each group  Ethnicity - White, Hispanic, African American | 38-86 | Pre test-Post test repeated measures Randomised pilot study | Night Monitoring System | 50  (No allocation concealment or blinding, high number of dropouts) | 1. System reliability.  2. Satisfaction with the NMS.  3. Nighttime injuries.  4. Unattended exits from home.  5. Mechanisms to manage nightime activity.  6. Baseline variables including short version of Zarit Burden Interview; The neuropsychiatric Inventory | 1 - 5 = No.  6. Zarit Burden Interview = Yes;  Quebec User Evaluatio of Satisfaction with Assistive Technology Questionnaire = Yes | - AT is adjunct to caregiving. Experimental group 85% less likely to sustain an event. - No significant differences in sleep diary variables. - Caregivers reported satisfaction and confidence in preventing night time injuries and exit. |
| 33 | Rowe MA et al ^33^  2010 | USA | 26/27 [Experiemental/control] – Spouses + Daughters + Sons  (6/3 Men; 18/22 Women).  Dropouts = 4 in each group  Ethnicity - White, Hispanic, African American | 38-86 | Pre test-Post test Controlled Clinical Trial | Night Monitoring System | 50  (No allocation concealment or blinding) | 1. Caregiver distress about night time activity  2. Sleep diary  3. actigraphy | 1. Generated for this study (10-point Likert-type scale.  2. Subjective 5-point scale  3. Analysis of sleep-wake cycles | - No significant improvement with sleep when NMS was used. - No quantifiable reduction in worry. - NMS not sufficient as standalone treatment |
| 34 | Olsson A et al^34^  2015 | Sweden | 3 – Spouses  (3 women) | 72-74 | Three - Experimental single case studies (A1B1A2B2) | Passive Positioning Alarm | 50  (convenience sample, researchers involved in data collection and provision of AT) | 1. Percentage of days with independent outdoor activities.  2. Spouse's worry concerning PwD's independent outdoor activities (1-10 not worried at all to extremely worried).  3. General well-being for spouses (0-10 not well-being at all to extremely good).  4. Caregiver burden scale (1 not at all to 4 often). | 1-3 = No;  4 = published for stroke patients | - Decreased level of worry about PwD's independent outdoor activities. - No significant changes in perceived well-being and burden. |
| 35 | Pot AM et al^35^  2012 | Netherlands | 33- Spouses + Children  (2 Men; 26 Women)  Incomplete = 5 | >63 | Pre test-Post test Trial | GPS tracking device | 60  (Researchers involved in data collection and provision of AT) | 1. overall global impression of device from 1 to 10.  2. Structured questions for use of the device from totally agree, totally disagreem agree and disagree.  3. Accepatability using Agree, neutral, disagree.  4. Self-perceived pressure from informal care scale | 1-3 = No;  4 = published | - 77% of carers would recommend use of GPS. - 30% carers reported more time for other things; use of GPS rated at 7.2/10. - Role overload feelings not significantly reduced. - Decrease in the feelings of worry when they could reach PwD. - Forgetting to take device was not a problem as carers mainly lived with PwD. |
| 36 | Kinney J M et al^36^  2004 | USA | 19 – Spouses + Son + Daughter + Sister + Great Niece.  (8 men; 11 women).  Incomplete = 6.  Ethnicity – European American, African American, Hispanic, Asian | 36-82 | Survey and exit interview | Smart home management system (Xanboo); cameras and sensors, cell phone | 40  (Incomplete reporting of outcomes, high detection and performance bias) | 1. Family obligation, competence, role captivity, loss of self, overload, expressive support in caregiving.  2. Retrospective time budget.  3. Exit interview questions | No | - Caregivers able to learn to use technology. - Majority indicated having the system made lives easier. - 11/16 report positive change in how they spend their time. - 3/3 individuals report a positive change in relationship with PwD. - Caregivers interested in continuing to use the system. - Technology needed constant monitoring, which made it difficult for carers. |
| 37 | Duff P et al^37^  2007 | Ireland, England, Finland, Lithuania  Norway | 127  (Gender not reported)  Incomplete = 47 | Not reported | Before and After Survey | Calendar, Lamp, Gas cooker monitor, locator, picture telephone, medicine reminder | 40  (sampling strategy unclear and detection bias unclear) | 1. Usefulness.  2. Levels of satisfaction.  3. Recommend device to a friend.  4. Willing to pay for device.  5. Carer wellbeing score | - 1. = No.   5 = Yes | - 63.5% used AT. - 77.8% felt the AT was useful for PwD. - 90.1% satisfied with AT. - 94.5% would recommend the device. - 81.3% willing to pay for device. - Slight decrease in overall level of carer burden during trial. - Calendar, cooker monitor and picture telephone gave most satisfaction and most useful. |
| 38 | Rialle V et al^38^  2008 | France | 350 – Spouses + Siblings + Relatives + Other  (89 men; 181 women)  Incomplete = 80 | 54-98 | Cluster sample survey | Smart home technologies - fall sensor; oral call identification; video surveillance; tracking device; robot care; video conferencing | 75  (unclear information on detection bias) | 1. Questionnaire in three sections  a. general information;  b. Current skills and practice and viewpoints on specific technologies;  c. Economical effort and support. (not at all, little, moderately, very much).  Included 'mini' Zarit score | No | - Among helpful devices, tracking devices most appreciated, especially by women and younger caregivers n=195,196 - Videoconferencing was second most appreciate. - Least appreciated was fall sensors inserted inside the body, under the skin. - Younger caregivers found technology more useful than elderly. |
| 39 | Landau R et al^39^  2009 | Germany and Israel | 69 – Spouses + Sons + Daughters + Other family members  (16 men; 53 women) | Not reported | Survey | GPS tracking device | 75  (convenience sample) | 1. Attitude towards use of electronic tracking questionnaire - 31 items on 4-point Likert scale from do not agree at all to very much agree.  2. Who should make decisions regarding use of GPS device - 4-point Likert scale. 3. Six Items adapted from Quebec User Evaluation of Satisfaction with Assistive Technology scale | No | - GPS device used for sake of patients' safety or for carers’ peace of mind. - Tracking device can be viewed as contrary to human dignity. - Tracking a PwD in the community is an intra-family issue. - Carers want 'user-friendly' device. |
| 40 | Chen Y-C et al^40^  2012 | Taiwan | 3 – Spouses  (1 man; 2 women) | 28-86 | Survey | Electronic GPS | 25  (convenience sample, high detection and performance bias) | 1. Lost seeking devices used.  2. Wandering behaviour.  3. Functions in lost seeking devices.  4. Faults in lost seeking devices.  5. Importance of the information  6. importance of purchase consideration. | No | - Computer skills of carers was important in deciding use of technology. - Acceptance of product. - Carers wanted products that are not easy to dismantle by PwD. |
| 41 | Alwin J et al^41^  2013 | Sweden | 47 – Spouses + Children  (16 men; 31 women) | 46-77 | Survey | Easy-to-use telephones, door alarms, alarms and emergency transmitters | 60  (Detection and performance bias unclear) | 1. Carers of Older People in Europe (COPE index).  2. Patient perspective on Care and Rehabilitation process instrument (POCR).  3. How do you value the significance of the intervention? (1= of no significance, 5= of greatest possible significance) | 1= published. 2 = Published but modified for this study.  3= No | - 33/47 (70%) perceived AT as having great significance - Alarms and safety AT may have a positive effect on sense of security for carers. - AT may not achieve major improvements but instead smaller changes that could lead to slight improvements of the situation. |
| 42 | Lim F S et al^42^  2013 | Australia | 24 – Family + Friend  (3 men; 21 women)  Dropouts = 3 | 34-85 | Before, during and after survey | iPad | 50  (Detection and performance bias unclear and some selective reporting) | 1. Previous experience of use of technology.  2. PwD using device independently.  3. Carer's perspectives on usefulness of the device. | No | - 48% carers reported they would use the iPad regularly with PwD. - 50% of PwD used device independently providing relief for carers. |
| 43 | McKenzie B et al^43^  2013 | USA | 60  (7 men; 53 women)  incomplete = 1 | Not reported | Before and after survey | Motion sensor with remote alarm, wireless camera with handheld liquid crystal display night vision, proximity range alarm, wireless home security system, medication organiser, locating technologies, bed occupancy sensor, touchpad key locks, window alerts, water detector alert | 80  (some selective reporting) | 1. Safety assessment Scale. 2. Caregiver Vigilance Scale.  3. Peace of Mind scale.  4. Sleep Disorders inventory | Yes | - Technology that was perceived as institutional was resisted. - New door locks were considered intrusive and resisted by all carers. - Medication organiser was most used. - AT provided immediate relief, reduced stress and helped carers provide care more easily and safely. |
| 44 | Schulz R et al^44^  2016 | USA | 512 – Spouses + Sons +Daughters + Other family members + Friends  (251 men; 261 women)  Incomplete= 75  Ethnicity – White, Hispanic, African American,  Asian | 18-64 | Survey | Emergency response system; sensors; | 50  (convenience sample) | 1. General technology attitudes -10-point scale. | No | - Carers willing to pay and are receptive to AT. - Combined government and private pay arrangements help disseminate AT. |
| 45 | Kamimura T^45^  2017 | Japan | 5 – Spouse + Daughter + Daughter-in-law  (Gender not reported) | 52-65 | Survey | Automatic Medication Dispenser | 25  (high detection and performance bias) | 1. Carer Burden (1=no burden; 2= little burden; 3=mild burden; 4=moderate to severe burden). | No | - Caregiver burden reduced compared to before device use. - caregivers-maintained score of little burden or less and one caregiver had a score of mild burden throughout. - Continued caregiver support is main factor for medication adherence using the device. |
| 46 | Korchut A et al^46^  2017 | Poland Spain | 81-  (26 men; 54 women)  incomplete = 1 | Not reported | Survey | Robotic assistants | 50  (Convenience sample) | 1. Social acceptance.  2. human-robot interaction | No | - AT needed for handling emergencies at home. - Reminders for medication was a high priority. - Carers viewed robotic technology positively. |
| 47 | Megges H et al^47^  2017 | Germany | 18 – Spouses + daughters  (10 men; 8 women)  Dropout = 1 | 41-78 | Before and after survey | Mobile locating system with GPS | 40  (high performance and detection bias) | 1. Zarit burden interview.  2. General self-efficacy scale.  3. User diary.  4. How satisfied they were with the prototype (1=not at all satisfied to 4= very satisfied) | 1,2 = Yes.  3,4=No | - No significant difference in caregiver burden or self-efficacy from T1 to T2. - 76.5% willing to purchase prototype. - Recommend using qualitative methods for further research. |

| Mixed Methods Studies | | | | | | | | | | |
| --- | --- | --- | --- | --- | --- | --- | --- | --- | --- | --- |
| No | Authors  Date | Country | Participants | Age range | Study Design | Assistive Technology | MMAT Appraisal (%) | Measures | Validated measures | Major Findings |
| 48 | Topo P et al^48^  2007 | Lithuania  Norway  Ireland  United Kingdom  Finland | 50 - Spouses + Children + Grandchildren + Friends.  (4 men; 21 women).  Incomplete = 25 | 29-84 | 1. Burden of care questionnaire  2. Interviews | Night and Day Calendar | 40  (High performance and detection bias and large dropout rate) | 1. The need for care and treatment. 2. Use of services and quality of life. 3. Greene's Relative Stress Scale | 1,2 = No.  3= Published | - Most carers benefitted if PwD used the NDC. - Important to balance expectations vs benefits from AT. - NDC benefits in mild to moderate stages of dementia. |
| 49 | Meiland F et al^49^  2012 | Netherlands  Ireland  Sweden | 41 – Spouses + Children  (25 men; 16 women) | 23-79 | Pre test-Post test Questionnaires  2. Semi-structured interviews.  3. Diary.  4. Observations | COGKNOW Day Navigator (CDN) - touchscreen, mobile device, home based sensors, actuators | 50  (high performance and detection bias, integration of qualitative and quantitative data unclear) | 1. Short Sense of Competence Questionnaire.  2. One question regarding overall judgement on QoL of the carer | 1= Yes.  2=No. | - Carers found time and day indication and reminders most useful. Carers dissatisfied with technical instability of the system. - No statistical significant difference in pre and post test scores on QoL or burden. - Carers and PwD willing to accept AT. |
| 50 | Nijhof N et al^50^  2013 | Netherlands | 16 – Spouses + Sons + Daughters  (6 men, 10 women) | 35-79 | 1. Log file of system use.  2. Interviews | PAL4 BV - Agenda for the day, diary, two-way video contact, memory games to play, Music and movies, information on dementia and the village they live in | 60  (High performance and detection bias) | 1. Log files - number of clicks and buttons pressed on the touch screen.  2. interviews on uptake and impact with carers | No | - Installation of AT was sometimes too late in the process of dementia. Higher functioning clients might find the system stigmatising. Agenda reminders was most used followed by video with family. - All considered the system useful and user friendly. Some elderly caregivers (n=3) used the system for their own reminders and to play games as well. - Did not reduce burden on caregiver but provided more time for self. - Carers had to spend extra time to learn to use the system. |
| 51 | Mehrabian S et al^51^  2014 | France | 30 – Spouses + Children  (10 men; 20 women) | 54-74 | 1. Survey  2. Semi-structured interviews. | Telecare system - sensors, videoconferencing, detecting emergencies, cognitive stimulation exercises. Medicines and task reminders | 25  (consideration of results in context is unclear and no clear integration of quantitative and qualitative data) | 1. Questionnaire on use and usefulness.  2. Interview questions | No | - No differences in answers between offspring and spouses groups and between men and women carers. - Carers who did not live with PwD replied system would make them feel more secure. - Carers found emergency detection system more useful than PwD. - Carers also perceived the system will give them more freedom to leave PwD alone; they perceived it would improve their quality of life. - Carers felt the system could allow PwD stay at home for as long as possible. - Spouses denied the need for help to care for PwD more than children. |
| 52 | Lewis V et al^52^  2015 | Australia | 59 – Spouses + Others  (16 men; 35 women).  Incomplete = 8 | 30-70 | 1. Pre test-Post test self-report questionnaire. 2. Diary.  3. Semi-structured phone interviews. | MP3 player | 90  (theoretical framework for qualitative data analysis not reported) | 1. Kessler-10 measure of psychological distress -1 =none of the time to 5 = all of the time.  2. General health question-one item.  3. Life satisfaction questions - 1=very dissatisfied to 11=very satisfied. 4. Family caregiver self-efficacy scale - 1= not at all certain to 10 = very certain.  5. Caregiving and stress measure. 6. Self-care and healthy behaviours. | Yes | - Increase in symptom management and self-efficacy for the carer. - Reduced caregiver need for vigilance. - No significant changes in caregiver stress. |
| 53 | Hattink B J et al^53^  2016 | Germany  Netherlands  Belgium | 17/15 [Experiemental/Control] – Spouses + Children + Other  (7/6 men; 10/8 women)  Dropouts = 9/9 | 29-85 | 1. Pre test-Post test control group design with matched groups (Netherlands, Belgium). Randomised Controlled Trial (Germany).  2. Focus Groups | Integrated Rosetta system: Elderly Day Navigator + The early Detection System + Unattended autonomous surveillance system | 40  (Theoretical framework for qualitative data not reported, high detection, attrition and performance bias) | 1. Usefulness and user-friendliness. 2. Short sense of competence questionnaire. | 1 = No.  2 = yes | - The system was not always available due to technical faults and maintenance. - Carers found the system very useful (n=9 - all completers). - Carers described system as having a noticeable impact and offered a 'safer feeling' (n=3). - Carers experienced little less burden. - User friendliness not rated very high due to technical issues. - Concerns about privacy were occasionally raised. - No significant differences between control and experimental groups in outcome measures. |
| 54 | Navarro R F et al^54^  2016 | Mexico | 3 – Spouses + Daughter  (3 women) | 43-66 | 1. Questionnaires  2. Diary.  3. interviews | Assisted cognition system - touchscreen reminders for the day, current date and time, tracking | 40  (Theoretical framework for qualitative data not reported, high detection and performance bias) | 1. Caregiver burden - Zarit Burden Interview. 2. Caregiver self-efficacy - revised Scale for caregiving Self-Efficacy | Yes | - Carers report feelings of 'safety' assurance that medicines were managed on time. - Trend of decreasing carer burden (from moderate to mild) and increasing self-efficacy -developing skills to deal with problem behaviour - Carers also note decrease in level of constant attention required, leaving carers calmer and more relaxed. |
| 55 | Liu L et al^55^  2017 | Canada | 46 - Spouses + Children + Friend + Daughter-in-law  (11 men; 35 women) | 41-71 | 1. Pre test-Post test questionnaires2. Focus groups | GPS | 75  (Theoretical framework for qualitative data not reported) | 1. Questionnaire - 1=strongly disagree to 5 = strongly agree.  2. Zarit Burden Scale.  3. Focus groups. | No | - Caregiver social influence belief had a positive and significant impact in the intention to use the GPS in both carers and PwD. - Focus group - Giving PwD more independence, peace of mind, highly recommend use of the device. - Some problems relate to false alarms and notifications. |
| 56 | Tyack C et al^56^  2017 | England | 12  (2 men, 10 women)  Ethnicity – White British, White Irish | 48-77 | 1. Quasi-experimental repeated measures. Questionnaire. 2. Semi-structured interviews | Tablet Computer | 75  (High performance and detection bias) | 1. Quality of Life - Alzheimer's Disease (QoL-AD) scale.  2. Semi-structured interviews | Yes  Thematic analysis | - Beneficial effect on well-being of PwD as people completed more sessions. - Carers spent more time with PwD and related it as good. - Carers reappraised what PwD could do with the app more positively. |

References of included studies:

1. Altus DE, Mathews RM, Xaverius PK, Engelman KK, Nolan BAD. Evaluating an electronic monitoring system for people who wander. *Am J Alzheimer’s Dis*. 2000;15(2):121-125. doi:10.1177/153331750001500201

2. Cahill S, Begley E, Faulkner JP, Hagen I. Findings from Ireland on the use and usefulness of assistive technology for people with dementia. *Technol Disabil*. 2007;19(Volume 19, Number 2-3 / 2007):133-142.

3. Starkhammar S., Nygard L. Using a timer device for the stove: Experiences of older adults with memory impairment or dementia and their families. *Technol Disabil*. 2008;20:179-191. http://ovidsp.ovid.com/ovidweb.cgi?T=JS&PAGE=reference&D=emed8&NEWS=N&AN=2008505436.

4. Faucounau V, Riguet M, Orvoen G, et al. Electronic tracking system and wandering in Alzheimer’s disease: A case study. *Ann Phys Rehabil Med*. 2009;52(7-8):579-587. doi:10.1016/j.rehab.2009.07.034

5. Spring HJ, Rowe MA, Kelly A. Improving Caregivers’ Well-Being by Using Technology to Manage Nighttime Activity in Persons with Dementia. *Res Gerontol Nurs*. 2009;2(1):39-48. doi:10.3928/19404921-20090101-10

6. Landau R, Auslander GK, Werner S, Shoval N, Heinik J. Families and professional caregivers views of using advanced technology to track people with dementia. *Qual Health Res*. 2010;20(3):409-419. doi:10.1177/1049732309359171

7. Powell J, Gunn L, Lowe P, Sheehan B, Griffiths F, Clarke A. New networked technologies and carers of people with dementia: An interview study. *Ageing Soc*. 2010;30(6):1073-1088. doi:10.1017/S0144686X1000019X

8. White EB, Montgomeiy P, McShane R. Electronic tracking for people with dementia who get lost outside the home: A study of the experience of familial carers. *Br J Occup Ther*. 2010;73(4):152-159. doi:10.4276/030802210X12706313443901

9. Rosenberg L, Nygård L. Persons with dementia become users of assistive technology: A study of the process. *Dement*. 2012;11(2):135-154. doi:10.1177/1471301211421257

10. Olsson A, Engström M, Skovdahl K, Lampic C. My, your and our needs for safety and security: relatives’ reflections on using information and communication technology in dementia care. *Scand J Caring Sci*. 2012;26(1):104-112. doi:10.1111/j.1471-6712.2011.00916.x

11. Rosenberg L, Kottorp A, Nygård L. Readiness for Technology Use With People With Dementia: The Perspectives of Significant Others. *J Appl Gerontol*. 2012;31(4):510-530. doi:10.1177/0733464810396873

12. Martin S, Augusto JC, Mccullagh P, et al. Participatory research to design a novel telehealth system to support the night-time needs of people with dementia: NOCTURNAL. *Int J Environ Res Public Health*. 2013;10(12):6764-6782. doi:10.3390/ijerph10126764

13. Nijhof N, van Gemert-Pijnen LJ, Woolrych R, Sixsmith A. An evaluation of preventive sensor technology for dementia care. *J Telemed Telecare*. 2013;19(2):95-100. doi:10.1258/jtt.2012.120605

14. Olsson A, Engström M, Lampic C, Skovdahl K. A passive positioning alarm used by persons with dementia and their spouses - A qualitative intervention study. *BMC Geriatr*. 2013;13(1). doi:10.1186/1471-2318-13-11

15. Riikonen M, Paavilainen E, Salo H. Factors supporting the use of technology in daily life of home-living people with dementia. *Technol Disabil*. 2013;25(4):233-243. doi:10.3233/TAD-130393

16. Hastall MR, Eiermann ND, Ritterfeld U. Formal and informal carers’ views on ICT in dementia care: Insights from two qualitative studies. *Gerontechnology*. 2014;13(1):53-58. doi:10.4017/gt.2014.13.1.003.00

17. Jentoft R, Holthe T, Arntzen C. The use of assistive technology in the everyday lives of young people living with dementia and their caregivers. Can a simple remote control make a difference? *Int Psychogeriatrics*. 2014;26(12):2011-2021. doi:10.1017/S1041610214001069

18. Meiland FJM, Hattink BJJ, Overmars-Marx T, et al. Participation of end users in the design of assistive technology for people with mild to severe cognitive problems; the European Rosetta project. *Int Psychogeriatrics*. 2014;26(5):769-779. doi:10.1017/S1041610214000088

19. Milne H, van der Pol M, McCloughan L, et al. The use of global positional satellite location in dementia: A feasibility study for a randomised controlled trial. *BMC Psychiatry*. 2014;14(1):1-13. doi:10.1186/1471-244X-14-160

20. Burstein AA, DaDalt O, Kramer B, D’Ambrosio LA, Coughlin JF. Dementia caregivers and technology acceptance: Interest outstrips awareness. *Gerontechnology*. 2015;14(1):45-56. doi:10.4017/gt.2015.14.1.005.00

21. Gibson G, Dickinson C, Brittain K, Robinson L. The everyday use of assistive technology by people with dementia and their family carers: A qualitative study. *BMC Geriatr*. 2015;15(1):1-10. doi:10.1186/s12877-015-0091-3

22. Arntzen C, Holthe T, Jentoft R. Tracing the successful incorporation of assistive technology into everyday life for younger people with dementia and family carers. *Dementia*. 2016;15(4):646-662. doi:10.1177/1471301214532263

23. Grigorovich A, Rittenberg N, Dick T, et al. Roles and coping strategies of sons caring for a parent with dementia. *Am J Occup Ther*. 2016;70(1):1-9. doi:10.5014/ajot.2016.017715

24. Newton L, Dickinson C, Gibson G, Brittain K, Robinson L. Exploring the views of GPs, people with dementia and their carers on assistive technology: A qualitative study. *BMJ Open*. 2016;6(5):1-8. doi:10.1136/bmjopen-2016-011132

25. Ekström A, Ferm U, Samuelsson C. Digital communication support and Alzheimer’s disease. *Dementia*. 2017;16(6):711-731. doi:10.1177/1471301215615456

26. Evans N, Collier L. An exploration of the experience of using calendar reminders for people with dementia and family carers. *Dementia*. 2017:1471301217734916. doi:https://dx.doi.org/10.1177/1471301217734916

27. Hassan L, Swarbrick C, Sanders C, et al. Tea, talk and technology: patient and public involvement to improve connected health ‘wearables’ research in dementia. *Res Involv Engagem*. 2017;3(1):12. doi:10.1186/s40900-017-0063-1

28. Holthe T, Jentoft R, Arntzen C, Thorsen K. Benefits and burdens: family caregivers’ experiences of assistive technology (AT) in everyday life with persons with young-onset dementia (YOD). *Disabil Rehabil Assist Technol*. September 2017:1-9. doi:10.1080/17483107.2017.1373151

29. Lorenz K, Freddolino PP, Comas-Herrera A, Knapp M, Damant J. Technology-based tools and services for people with dementia and carers: Mapping technology onto the dementia care pathway. *Dementia*. February 2017:147130121769161. doi:10.1177/1471301217691617

30. Wang RH, Sudhama A, Begum M, Huq R, Mihailidis A. Robots to assist daily activities: Views of older adults with Alzheimer’s disease and their caregivers. *Int Psychogeriatrics*. 2017;29(1):67-79. doi:10.1017/S1041610216001435

31. Gitlin LN, Winter L, Dennis MP. Assistive devices caregivers use and find helpful to manage problem behaviors of dementia. *Gerontechnology*. 2010;9(3):408-414. doi:https://dx.doi.org/10.4017/gt.2010.09.03.006.00

32. Rowe MA, Annette K, Horne C, et al. Reducing Dangerous Nighttime Events in Persons with Dementia Using a Nighttime Monitoring System. *Alzheimers Dement*. 2009;5(5):419-426. doi:10.1016/j.jalz.2008.08.005

33. Rowe MA, Kairalla JA, McCrae CS. Sleep in dementia caregivers and the effect of a nighttime monitoring system. *J Nurs Scholarsh*. 2010;42(3):338-347. doi:10.1111/j.1547-5069.2010.01337.x

34. Olsson A, Engström M, Åsenlöf P, Skovdahl K, Lampic C. Effects of Tracking Technology on Daily Life of Persons with Dementia: Three Experimental Single-Case Studies. *Am J Alzheimers Dis Other Demen*. 2015;30(1):29-40. doi:10.1177/1533317514531441

35. Pot AM, Willemse BM, Horjus S. A pilot study on the use of tracking technology: Feasibility, acceptability, and benefits for people in early stages of dementia and their informal caregivers. *Aging Ment Heal*. 2012;16(1):127-134. doi:10.1080/13607863.2011.596810

36. Kinney JM, Kart CS, Murdoch LD, Conley CJ. Striving to Provide Safety Assistance for Families of Elders:The SAFE House Project. *Dementia*. 2004;3(3):351-370. doi:10.1177/1471301204045165

37. Duff P, Dolphin C. Cost-benefit analysis of assistive technology to support independence for people with dementia – Part 2: Results from employing the ENABLE cost-benefit model in practice. *Technol Disabil*. 2007;19:79-90.

38. Rialle V, Ollivet C, Guigui C, Hervé C. What Do family caregivers of Alzheimer’s disease patients desire in smart home technologies? Contrasted results of a wide survey. *Methods Inf Med*. 2008;47(1):63-69. doi:10.3414/ME9102

39. Landau. Attitudes of family and professional care givers towards the use of GPS for tracking tracking patients with dementia: a exploratory study. *Britsh J Soc Work*. 2009;39(4):92-670. doi:10.1093/bjsw/bcp037

40. Chen Y-C, Leung C-Y. Exploring functions of the lost seeking devices for people with dementia. *Work*. 2012;41:3093-3100. doi:10.3233/WOR-2012-0568-3093

41. Alwin J, Persson J, Krevers B. Perception and significance of an assistive technology intervention - the perspectives of relatives of persons with dementia*. *Disabil Rehabil*. 2013;35(18):1519-1526. doi:10.3109/09638288.2012.743603

42. Lim FS, Wallace T, Luszcz MA, Reynolds KJ. Usability of tablet computers by people with early-stage dementia. *Gerontology*. 2013;59(2):174-182. doi:10.1159/000343986

43. McKenzie B, Bowen ME, Keys K, Bulat T. Safe Home Program: A Suite of Technologies to Support Extended Home Care of Persons With Dementia. *Am J Alzheimer’s Dis Other Dementias*. 2013;28(4):348-354. doi:10.1177/1533317513488917

44. Schulz R, Beach SR, Matthews JT, Courtney K, De Vito Dabbs A, Mecca LP. Caregivers’ Willingness to Pay for Technologies to Support Caregiving. *Gerontologist*. 2016;56(5):817-829. doi:10.1093/geront/gnv033

45. Kamimura T. Older Adults with Alzheimer’s Disease Who Have Used an Automatic Medication Dispenser for 3 or More Years. *Clin Gerontol*. 2017:1-7. doi:https://dx.doi.org/10.1080/07317115.2017.1347594

46. Korchut A, Szklener S, Abdelnour C, et al. Challenges for service robots-requirements of elderly adults with cognitive impairments. *Front Neurol*. 2017;8(JUN):1-12. doi:10.3389/fneur.2017.00228

47. Megges H, Freiesleben SD, Jankowski N, Haas B, Peters O. Technology for home dementia care: A prototype locating system put to the test. *Alzheimer’s Dement (New York, N Y)*. 2017;3(3):332-338. doi:https://dx.doi.org/10.1016/j.trci.2017.04.004

48. Topo P, Saarikalle K, Begley E, Cahillb S, Holthe T, Macijauskiene J. “I don’t know about the past or the future, but today it’s Friday” - Evaluation of a time aid for people with dementia. *Technol Disabil*. 2007;19(2-3):121-131. http://ovidsp.ovid.com/ovidweb.cgi?T=JS&CSC=Y&NEWS=N&PAGE=fulltext&D=emed11&AN=46655527%5Cnhttp://sfx.nottingham.ac.uk:80/sfx_local?genre=article&atitle=%22I+don%27t+know+about+the+past+or+the+future%2C+but+today+it%27s+Friday%22+-+Evaluation+of+a+time+ai.

49. Meiland FJM, Bouman AIE, Sävenstedt S, et al. Usability of a new electronic assistive device for community-dwelling persons with mild dementia. *Aging Ment Heal*. 2012;16(5):584-591. doi:10.1080/13607863.2011.651433

50. Nijhof N, van Gemert-Pijnen JEWC, Burns CM, Seydel ER. A personal assistant for dementia to stay at home safe at reduced cost. *Gerontechnology*. 2013;11(3):469-479. doi:10.4017/gt.2013.11.3.005.00

51. Mehrabian S, Extra J, Wu YH, Pino M, Traykov L, Rigaud AS. The perceptions of cognitively impaired patients and their caregivers of a home telecare system. *Med Devices Evid Res*. 2014;8:21-29. doi:10.2147/MDER.S70520

52. Lewis V, Bauer M, Winbolt M, Chenco C, Hanley F. A study of the effectiveness of MP3 players to support family carers of people living with dementia at home. *Int Psychogeriatrics*. 2015;27(3):471-479. doi:10.1017/S1041610214001999

53. Hattink BJJ, Meiland FJM, Overmars-Marx T, et al. The electronic, personalizable Rosetta system for dementia care: exploring the user-friendliness, usefulness and impact. *Disabil Rehabil Assist Technol*. 2016;11(1). doi:10.3109/17483107.2014.932022

54. Navarro RF, Rodrï¿½guez MD, Favela J. Use and Adoption of an Assisted Cognition System to Support Therapies for People with Dementia. *Comput Math Methods Med*. 2016;2016(1):1-10. doi:10.1155/2016/1075191

55. Liu L, Miguel Cruz A, Ruptash T, Barnard S, Juzwishin D. Acceptance of Global Positioning System (GPS) Technology Among Dementia Clients and Family Caregivers. *J Technol Hum Serv*. 2017;35(2):99-119. doi:10.1080/15228835.2016.1266724

56. Tyack C, Camic PM, Heron MJ, Hulbert S. *Viewing Art on a Tablet Computer: A Well-Being Intervention for People with Dementia and Their Caregivers*. Vol 36.; 2017. doi:10.1177/0733464815617287
